# The Infrabranchial Musculature and Its Bearing on the Phylogeny of Percomorph Fishes (Osteichthyes: Teleostei)

**DOI:** 10.1371/journal.pone.0110129

**Published:** 2014-10-13

**Authors:** Aléssio Datovo, Mário C. C. de Pinna, G. David Johnson

**Affiliations:** 1 Museu de Zoologia da Universidade de São Paulo, Laboratório de Ictiologia, São Paulo, São Paulo, Brazil; 2 Division of Fishes, MRC 159, Department of Vertebrate Zoology, National Museum of Natural History, Smithsonian Institution, Washington, DC, United States of America; Sars International Centre for Marine Molecular Biology, Norway

## Abstract

The muscles serving the ventral portion of the gill arches ( = infrabranchial musculature) are poorly known in bony fishes. A comparative analysis of the infrabranchial muscles in the major percomorph lineages reveals a large amount of phylogenetically-relevant information. Characters derived from this anatomical system are identified and discussed in light of current hypotheses of phylogenetic relationships among percomorphs. New evidence supports a sister-group relationship between the Batrachoidiformes and Lophiiformes and between the Callionymoidei and Gobiesocoidei. Investigated data also corroborate the existence of two monophyletic groups, one including the Pristolepididae, Badidae, and Nandidae, and a second clade consisting of all non-amarsipid stromateiforms. New synapomorphies are proposed for the Atherinomorphae, Blenniiformes, Lophiiformes, Scombroidei (including Sphyraenidae), and Gobiiformes. Within the latter order, the Rhyacichthyidae and Odontobutidae are supported as the successive sister families of all remaining gobiiforms. The present analysis further confirms the validity of infrabranchial musculature characters previously proposed to support the grouping of the Mugiliformes with the Atherinomorphae and the monophyly of the Labriformes with the possible inclusion of the Pholidichthyiformes. Interestingly, most hypotheses of relationships supported by the infrabranchial musculature have been advanced by preceding anatomists on the basis of distinct data sources, but were never recovered in recent molecular phylogenies. These conflicts clearly indicate the current unsatisfactory resolution of the higher-level phylogeny of percomorphs.

## Introduction

**Figure 1:**
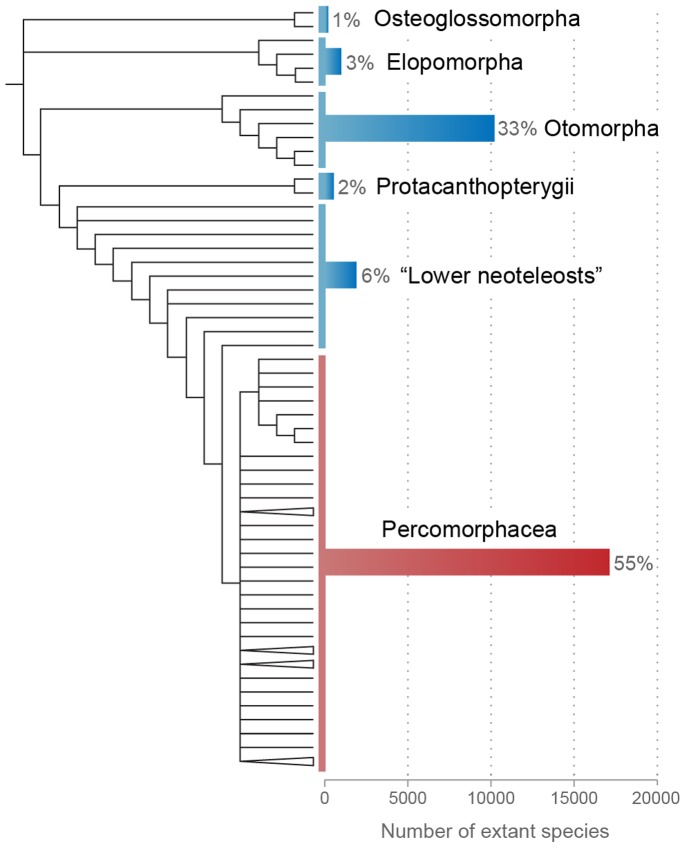
Phylogenetic relationships and species diversity of major extant teleostean groups. Cladogram based on Wiley and Johnson [1] with terminal branches representing orders; species diversity based on Eschmeyer and Fong [2].

**Figure 2:**
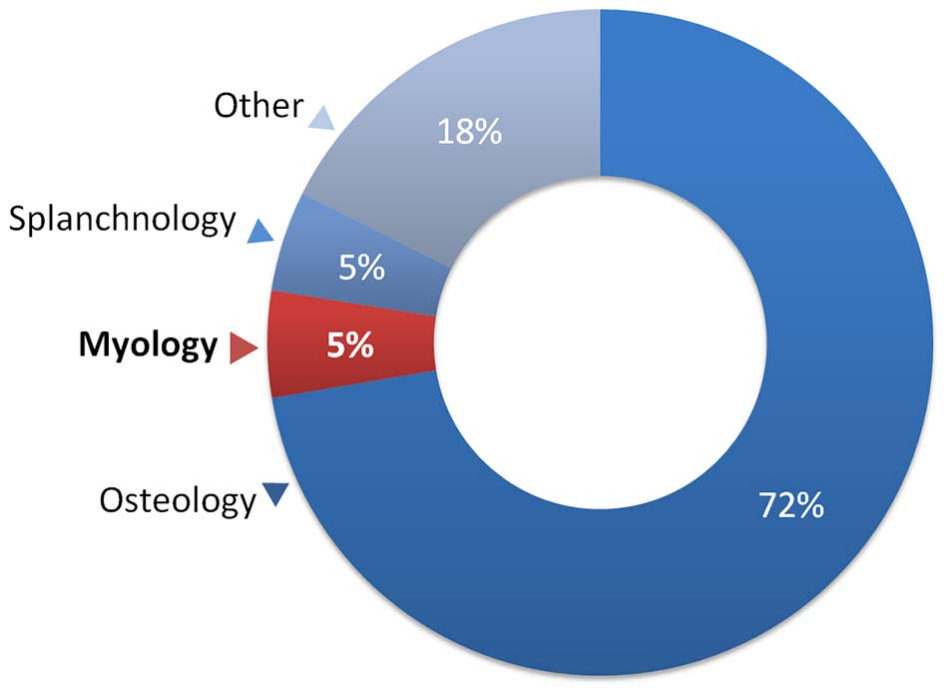
Categories of morphological synapomorphies delimiting the 50 major monophyletic groups of Percomorphacea. Synapomorphies and classification based on Wiley and Johnson [1].

**Figure 3:**
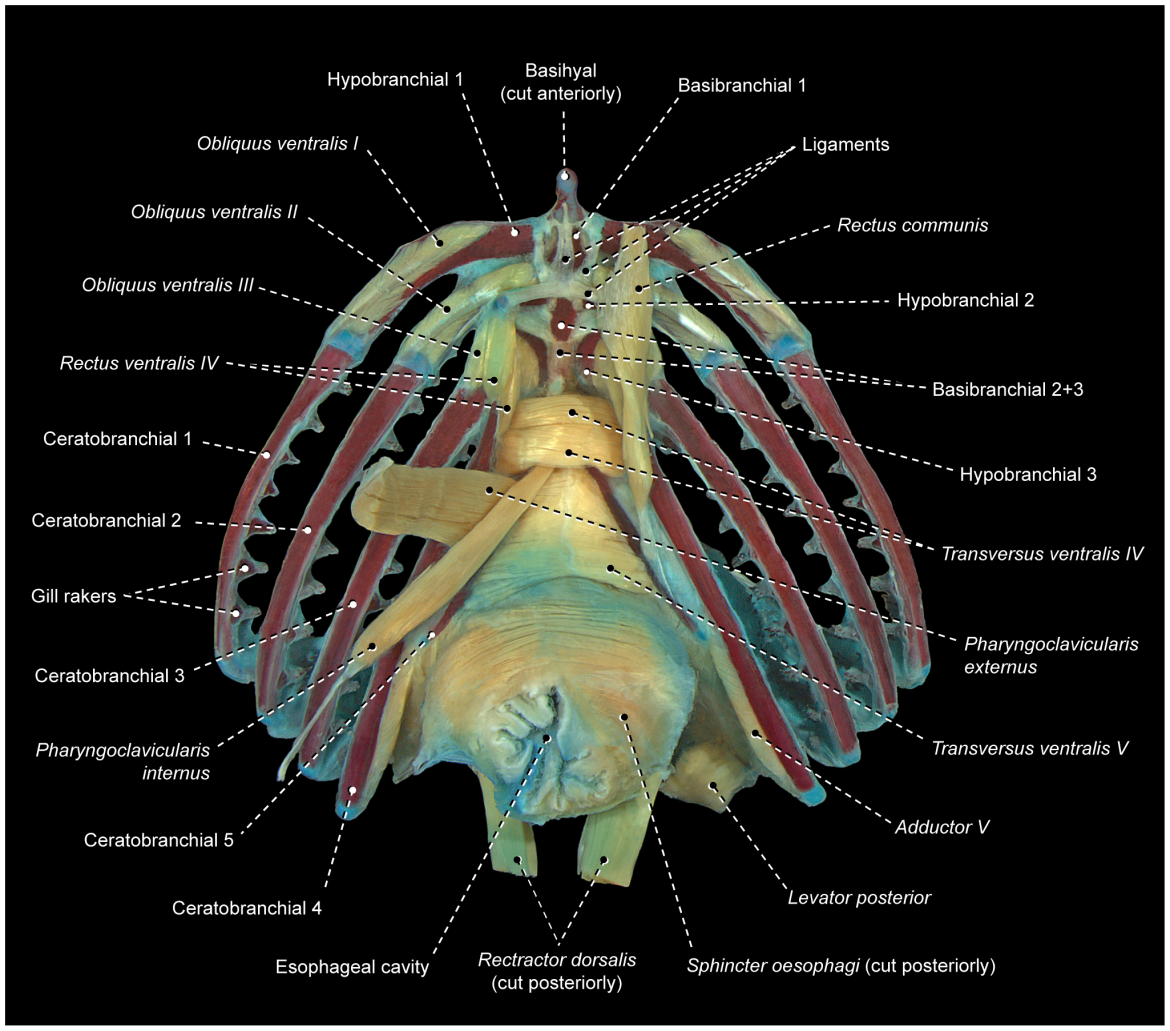
Branchial musculoskeletal system of *Scorpaena plumieri* (Scorpaeniformes: Scorpaenidae). MZUSP 67283. Ventral view; left *pharyngoclaviculares externus* and *internus*, and right *rectus communis* removed.

The division Percomorphacea (*sensu* Wiley and Johnson [1]) encompasses more than half of all species of living teleostean fishes and one quarter of extant vertebrates (Fig. 1; [2], [3]. In the recent classification of Wiley and Johnson [1], based on morphological evidence, ca. 17,000 extant species of percomorphs are distributed into 30 orders, 23 of which are placed in a vast unresolved basal polytomy (Fig. 1). Resolution of the interrelationships among the major percomorph groups remains one of the most daunting challenges in the systematics of the Teleostei. While several recent molecular studies pursued the problem [4]–[9], few comparative anatomical studies across a broad range of the Percomorphacea have been performed in the last decade [10].

While understanding of the intrarelationships of percomorphs is undoubtedly deficient, it is also true that several morphological complexes in the group, especially those from soft anatomy, remain severely under-studied. Among the morphological synapomorphies for the major percomorph groups listed in Wiley and Johnson [1], only 5% are from the skeletal musculature (Fig. 2). Despite important contributions to the knowledge of percomorph myology [10], definition of the major groups within this clade is still massively based on osteological characters (Fig. 2).

The branchial skeleton of fishes has been extensively studied by systematists especially after a series of studies by G. Nelson on that system [11]–[18]. More recently, Springer and Johnson [10] published a seminal monograph focusing on the dorsal and some posteroventral branchial muscles of bony fishes. In contrast, most muscles serving the ventral portion of the branchial arches have received scant attention from systematists ([19]: p. 294). To facilitate communication, the muscles associated with the ventral skeletal elements of the gill arches (basibranchials, hypobranchials, and ceratobranchials) are hereafter denominated infrabranchial muscles (from Latin *infra* meaning below, under). Data on the infrabranchial musculature of teleosts is sparse in the literature, with most studies on the subject dealing with only a single or a few closely related species [20]–[30].

This study presents a comparative analysis of the infrabranchial musculature of 90 representatives of all 30 percomorph orders. Our aim is to provide a first overview of the main patterns of variation in the infrabranchial musculature of percomorphs, a subject so far nearly unexplored. Although the taxonomic density of our sampling can and should eventually be increased, it is broad enough to provide a robust comparative framework that demonstrates the existence of significant phylogenetically-correlated information previously unreported, representing a number of new characters bearing on the higher-level phylogeny of the Percomorphacea. We expect our results can lay the foundations for further explorations into the infrabranchial myology of the group.

## Materials and Methods

Higher-level classification of the Teleostei follows Wiley and Johnson [1]. Families not listed in that study are as recognized in Eschmeyer and Fong [2]. Current taxonomic validity of the cited species follows Eschmeyer [31].

The research employed only ethanol-preserved specimens deposited in museums and did not involve animal experimentation or examination of fossil specimens. No permits were required for the described study, which complied with all relevant regulations. Preserved specimens were double-stained for cartilage and bone prior to dissection following the protocol of Datovo and Bockmann [32]. Examined material is listed in Table S1 and is deposited in the following institutions: Humboldt State University Fish Collection, USA (HSU); Laboratório de Ictiologia de Ribeirão Preto, Universidade de São Paulo, Brazil (LIRP); Museum of Comparative Zoology, Harvard University, USA (MCZ); Museu de Zoologia da Universidade de São Paulo, Brazil (MZUSP); Scripps Institution of Oceanography, USA (SIO); and National Museum of Natural History, Smithsonian Institution, USA (USNM). Access to material of those collections was authorized by respective curators. Specimens were examined at their original institutions or loaned to MZUSP or USNM between 01 May 2011 and 03 March 2014.

The term insertion refers to the attachment of the muscle to the structure (usually a bone) that presumably moves (or moves more intensely) during its contraction; origin is defined as the opposite muscle attachment to the stationary (or less movable) skeletal element [33]. Musculous attachment (origin or insertion) is when the muscles fibers attach directly to the skeleton without the mediation of any macroscopically evident tendon. In the tendinous attachment, the muscle fibers converge onto a macroscopically evident tendon, which, in turn, attaches to the skeleton. In some instances, the attachment of a muscle is partially musculous and partially tendinous.

Nomenclatures for the branchial musculature and skeleton follow Winterbottom [33] and Nelson [11], respectively.

Photographs were taken with a Leica DFC420 digital camera device attached to a Leica MZ16 stereomicroscope. Final images are composite multifocal montages derived from the stacking of several individual photographs taken at different focal planes. The multifocal montage was prepared in CombineZP [34]. Montages were then retouched digitally in Adobe Photoshop CS6 in order to remove artefacts (bubbles, specks, *etc.*) and chromatic imperfections in the original anatomical preparations (*e.g.*, muscles unduly stained with Alizarin red or Alcian blue) and to enhance structures of interest via adjustments in contrast, shadows and channel levels.

## Results and Discussion

### Generalized acanthopterygian pattern of the infrabranchial musculature

This section provides an overview of the general pattern of the infrabranchial musculature present in the majority of percomorph lineages. This pattern is also shared by examined representatives of other acanthopterygian orders (*i.e.*, Stephanoberyciformes, Zeiformes, and Beryciformes) and is thus interpreted as being plesiomorphic for the Percomorphacea. The generalized acanthopterygian pattern is presented as a baseline for the description and discussion of variations specific to smaller subgroups.

*Obliqui ventrales I*, *II*, and *III* are bilaterally-paired muscles sequentially arranged from first to third branchial arches, respectively (Fig. 3). Each *obliquus ventralis* has a broad origin on the ventral surface of the hypobranchial and a smaller area of insertion on the anteroventral region of the ceratobranchial of the same arch.

*Rectus ventralis IV* originates from the ventral face of hypobranchial 3 and inserts on the anteroventral portion of ceratobranchial 4 (Fig. 3).

**Figure 4:**
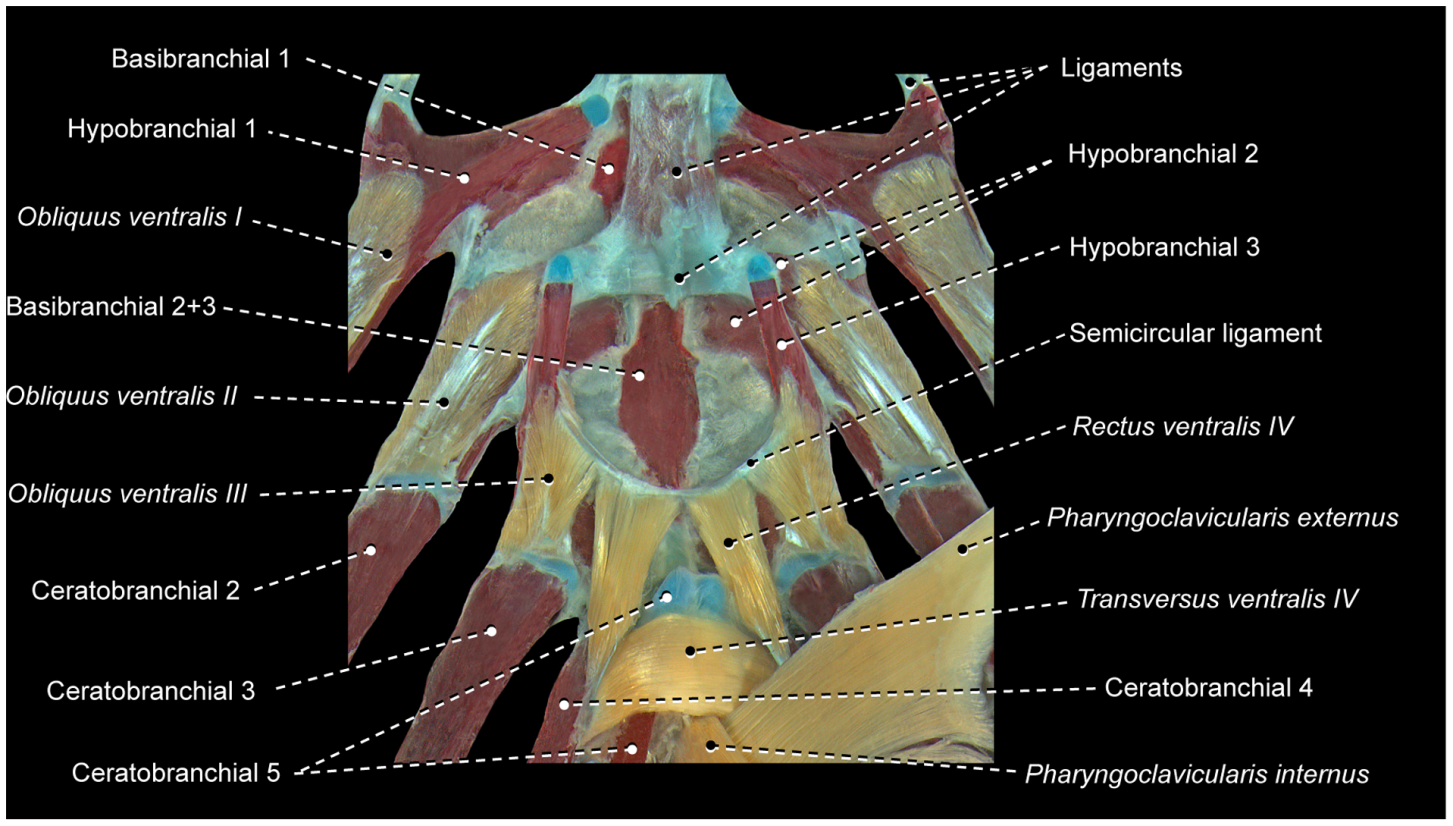
Semicircular ligament and associated structures of *Prionotus* sp. (Scorpaeniformes: Triglidae). MZUSP 71680. Ventral view; *rectus communis* removed.

The muscle fibers of *obliquus ventralis III* and *rectus ventralis IV* may attach directly to hypobranchial 3 or originate from this bone via a semicircular ligament (Fig. 4). When present, this ligament encircles posteriorly the anterior portion of the ventral aorta, which splits into left and right branches after its passage through the semicircular ligament. The presence of the semicircular ligament is likely plesiomorphic for percomorphs [10], [35]. However, both the presence of this ligament and its association with *rectus ventralis IV* and *obliquus ventralis III* exhibit a mosaic distribution across the Euteleosteomorpha (pers. obs., [10]), so that the precise phylogenetic interpretation of these features remains uncertain. In some instances, the sagittal portion of the semicircular ligament is attached to the basibranchials and this condition may confound the unequivocal recognition of that ligament.

*Transversi ventrales IV* and *V* are unpaired muscles that interconnect the anteroventral regions of the contralateral ceratobranchials 4 and 5, respectively (Fig. 3). Either or both muscles may exhibit a sagittal raphe. In all examined lower acanthopterygians and most percomorphs, *rectus ventralis V* has a sagittal raphe and, consequently, this condition is hypothesized to be plesiomorphic for percomorphs. Non-acanthopterygian percomorphs either have or lack a sagittal raphe in *rectus ventralis IV*, so that the polarity of this character is uncertain.

The *rectus communis* originates from the urohyal and inserts via a tendon on the lateral aspects of ceratobranchial 5 (Fig. 3). The anterior portion of the muscle is partially continuous with the *sternohyoideus*, which runs from the urohyal to the cleithrum. Lauder [28] proposed that the total or partial association of the *rectus communis* with the urohyal is a synapomorphy for the Ctenosquamata, and Stiassny [36] reported that this muscle has completely lost its association with hypobranchial 3 in holacanthopterygians (including percomorphs). However, a *rectus communis* with partial or total origin on hypobranchial 3 is present in most non-percomorph acanthopterygians and a few percomorphs herein examined. As a consequence, it is unclear whether or not the attachment of the *rectus communis* to hypobranchial 3 is plesiomorphic for percomorphs (see also “Modifications of unclear systematic value” below).

The *pharyngoclavicularis externus* originates from the anteroventral region of the cleithrum and inserts onto the anterolateral aspects of the ventral face of ceratobranchial 5 (Fig. 3). Both the origin and insertion of the muscle are primitively musculous.

The *pharyngoclavicularis internus* arises from the posterior portion of the anterodorsal face of the cleithrum and inserts on the anteroventral region of ceratobranchial 5 (Fig. 3). Near its insertion, the muscle passes medial to the *pharyngoclavicularis externus*.

The *sternohyoideus* originates from the anteroventral portion of the cleithrum, coracoid, and the myoseptum shared with the anteriormost segment of the *obliquus inferioris* ( = ventral division of the *hypaxialis*). The *sternohyoideus* is often segmented into three myomeres and inserts anteriorly onto the urohyal. A vertical membranous wall of connective tissue arises from the sagittal plane of the *sternohyoideus* and attaches dorsally to the ventral surface of the gill arches, thus dividing the branchial chamber beneath the arches into left and right halves. A strengthened oblique band of collagen embedded into the body of this membrane is often present between hypobranchial 3 and the dorsomedial portion of the *sternohyoideus*. The degree of differentiation of this strengthened band greatly varies across the examined acanthopterygians and the well-differentiated band of some taxa serves as an additional insertional tendon connecting the *sternohyoideus* to hypobranchial 3.

The *sphincter oesophagi* is an unpaired muscle that encircles the anterior portion of the esophagus and attaches anteriorly to ceratobranchial 5 and epibranchial 4 (Fig. 3).

Among percomorphs, the basic infrabranchial muscular configuration described above is present at least in the generalized members of the Cottiformes [37], [38], Gasterosteiformes [26], Nototheniiformes, Ophidiiformes [27], Scorpaenoidei [39], [40], Serranoidei [12], and several perciform families – Apogonidae, Centrarchidae [41], Haemulidae, Lutjanidae, Percidae [28], [29], Sciaenidae [42], [43], and Sparidae [30].

Several modifications in the generalized acanthopterygian pattern of the infrabranchial musculature arose in different percomorph lineages. These changes are described and discussed below in light of current hypotheses of phylogenetic relationships of percomorphs. Discussions of putative synapomorphies for the investigated groups are preceded by sequential numbers in bold type. Derived conditions that are apparently autapomorphic for single examined species or other lower taxonomic levels are outside the scope of this paper and are not included.

### Badidae, Nandidae, and Pristolepididae

**Figure 5:**
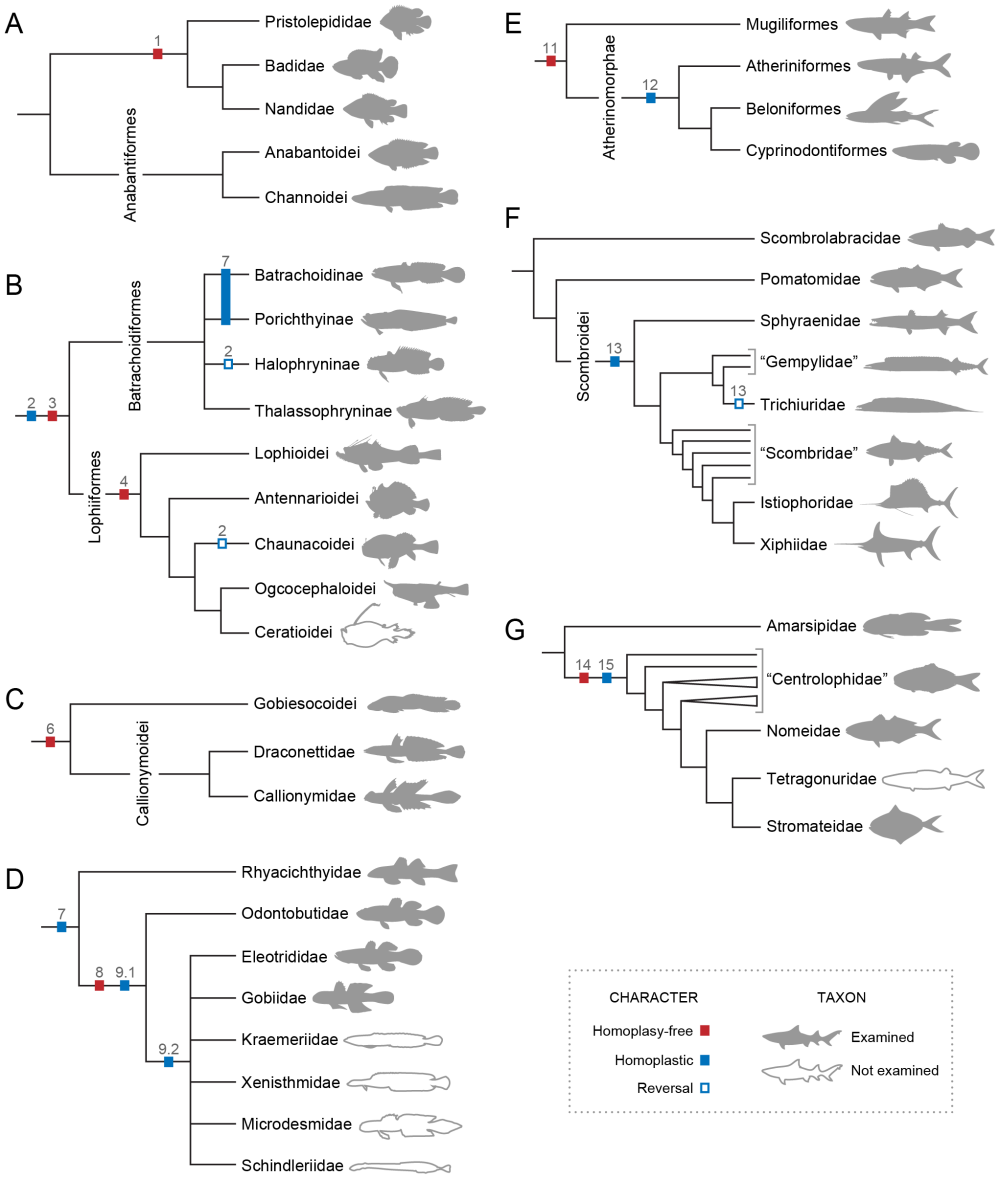
Maximum parsimony optimization (ACCTRAN) of the identified derived characters of the infrabranchial musculature. Characters superimposed on cladograms of the relationships among the (A) Anabantomorpha [8], [117]; (B) Batrachoidiformes and Lophiiformes [53], [54], [63]; (C) Callionymoidei and Gobiesocoidei [44], [66]–[68]; (D) Gobiiformes [76]–[78]; (E) Mugiliformes and Atherinomorphae [81], [95]; (F) Scombroidei, Pomatomidae and Scombrolabracidae [98]; and (G) Stromateiformes [111]. Characters numbered as in the text.

Several morphologists have long suggested a hypothesis of close relationship between the Anabantoidei, Channoidei, Pristolepididae, Badidae, and Nandidae (Fig. 5A) [10], [11], [44]–[49]. Springer and Johnson [10] coined the term Anabantomorpha to refer to the group and its monophyly was recently corroborated in two recent molecular analyses [8], [50]. While the Anabantoidei and Channoidei are often considered sister taxa ( = Anabantiformes *sensu* Wiley and Johnson [1]; = Labyrinthici Regan [51]), the relationships among the Pristolepididae, Badidae, and Nandidae vary among different studies. These three families were grouped into a monophyletic lineage in Near et al. [8], an arrangement corroborated by the following specialization of the infrabranchial musculature (Fig. 5A).

**Figure 6:**
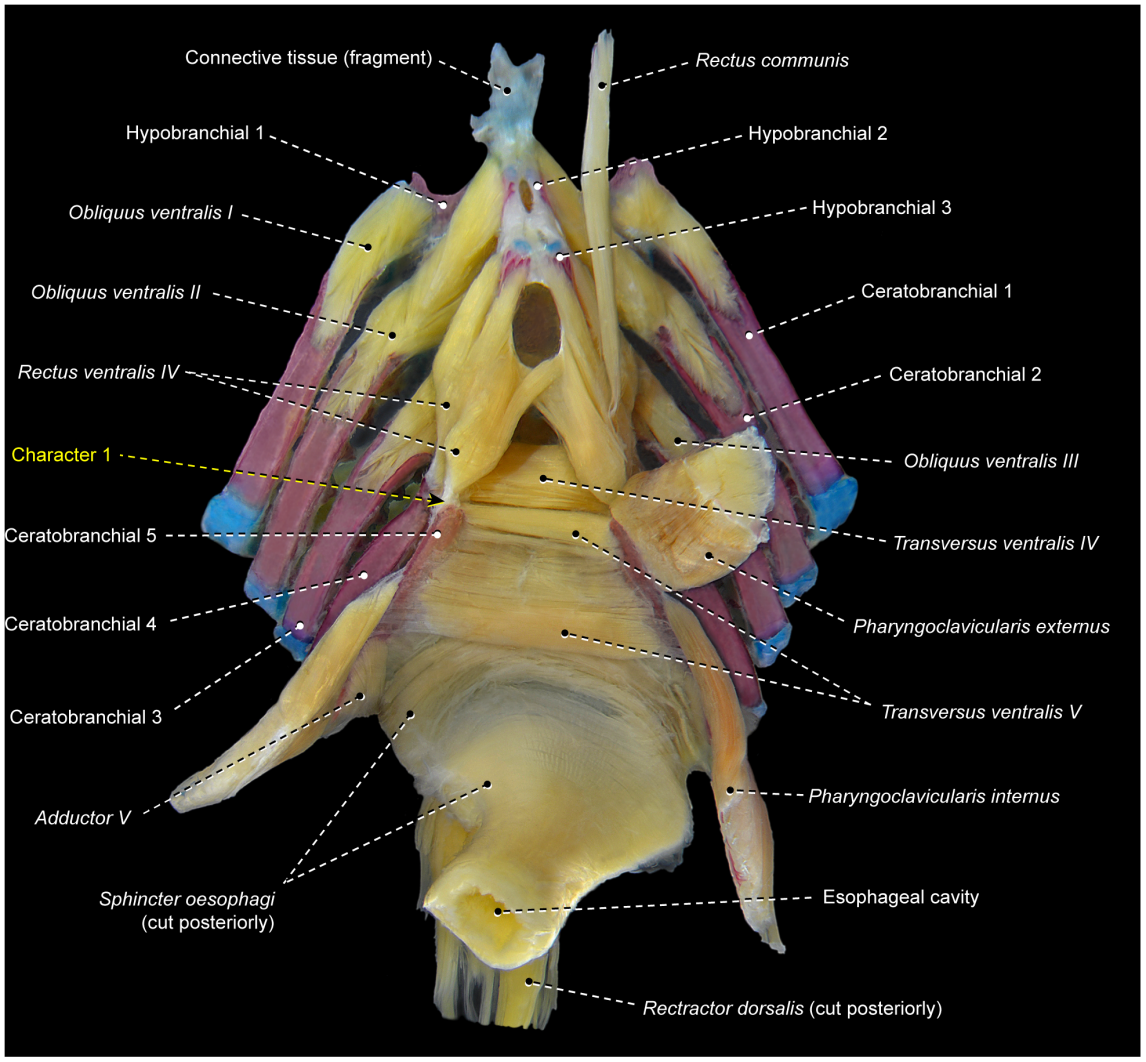
Branchial musculoskeletal system of *Pristolepis fasciata* (Perciformes: Pristolepididae). USNM 332697. Ventral view; gill rakers, right *pharyngoclavicularis externus* and *rectus communis* removed.

1 In the plesiomorphic condition for the Percomorphacea, *rectus ventralis IV* inserts solely on ceratobranchial 4 (Figs. 3, 4). In *Pristolepis*, *Badis*, and *Nandus* this muscle has an additional insertion on the anterior region of ceratobranchial 5 (Fig. 6). This derived condition is unique among the examined acanthopterygians and thus supports a pristolepidid-badid-nandid alignment (Fig. 5A).

### Batrachoidiformes and Lophiiformes

Several previous morphological studies proposed that the Batrachoidiformes and Lophiiformes form a monophyletic group, often termed Pediculati (Fig. 5B) [52]–[54]. This hypothesis has been repeatedly contested by molecular phylogenies that allocate these two orders in clades far removed from each other by several intercalated acanthopterygian taxa [8], [9], [55], [56]. The present study identified some unique derived myological characters shared by batrachoidiforms and lophiiforms.

**Figure 7:**
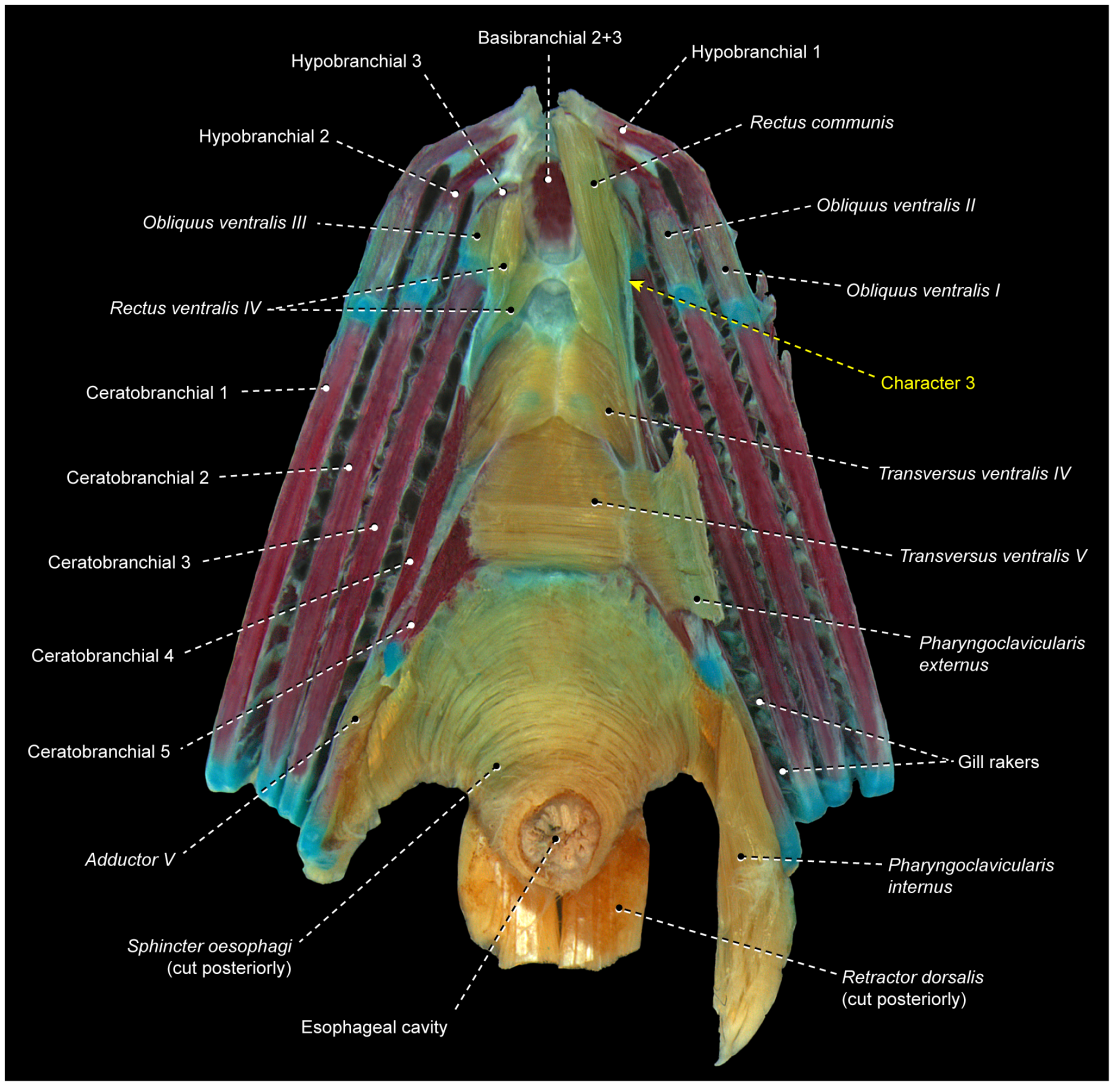
Branchial musculoskeletal system of *Porichthys porosissimus* (Batrachoidiformes: Batrachoididae). MZUSP 46971. Ventral view; right *pharyngoclavicularis externus*, *pharyngoclavicularis internus* and *rectus communis* removed.

2 Acanthopterygians primitively have a well-developed *obliquus ventralis I* muscle (Figs. 3, 4, 6). All lophiiforms and batrachoidiforms except *Chaunax* and *Porichthys* exhibit an extreme reduction of this muscle. *Obliquus ventralis I* is an almost imperceptible vestigial muscle in *Batrachoides* and *Antennarius*. The batrachoidiforms *Thalassophryne* and *Triathalassothia* and the lophiiforms *Lophius* and *Ogcocephalus* completely lack *obliquus ventralis I*.
3 Primitively in percomorphs, the *rectus communis* inserts solely on ceratobranchial 5 (Fig. 3). All examined batrachoidiforms and lophiiforms have a *rectus communis* with a medial section that retains the plesiomorphic insertion on ceratobranchial 5 and an additional lateral section that attaches to ceratobranchial 4 (Fig. 7). These sections are continuous with each other at their origins in batrachoidiforms and the lophiiforms *Antennarius* and *Chaunax*, whereas in *Lophius* and *Ogcocephalus* the two sections are completely separated along their entire extent. In batrachoidiforms the insertion of the *rectus communis* on ceratobranchial 4 is achieved via a tendon that partially merges dorsally with the insertional tendon of *rectus ventralis IV* (Fig. 7).

The two derived characters above clearly favor the hypothesis previously advanced by some morphologists that the Batrachoidiformes and Lophiiformes are sister taxa (Fig. 5B) [52]–[54]. Under that scheme, these characters are optimized as synapomorphies for the Pediculati, with reversals of character 2 in *Chaunax* and *Porichthys* (Fig. 5B) – it is worth mentioning that neither porichthyines nor chaunacids have been proposed as primitive members of their respective orders [8], [9], [55], [57], [58]. Most, but not all, batrachoidiforms and lophiiforms additionally share other specializations of the gill-arch system that are not found elsewhere among acanthopterygians, such as the extreme reduction of *obliquus ventralis II* (character 17), the musculous insertion of the *rectus communis* on ceratobranchial 5 (character 26), and the anterior tip of ceratobranchial 1 positioned posterior to that of ceratobranchial 2 (Fig. 7; Pietsch and Orr [59]: fig. 12B) – *vs.* anterior tip of ceratobranchial 1 anterior to that of ceratobranchial 2 (Fig. 3). However, the precise optimizations of such characters are problematic and demand a more encompassing taxonomic sampling of the Pediculati. On the molecular phylogenies of the Acanthopterygii [8], [9], [55], all foregoing similarities would be mapped as convergent in batrachoidiforms and lophiiforms. These studies grouped lophiiforms with tetraodontiforms and caproiforms, rather than with batrachoidiforms. Although some phenotypic traits were recently offered as possibly corroborating the alignment of lophiiforms with tetraodontiforms [60], [61], such a hypothesis definitely gains no support from the myological data herein investigated.

Monophyly of the Lophiiformes is supported to date by six synapomorphies [62], [63]. The following derived condition of the infrabranchial musculature is herein interpreted as an additional unique synapomorphy for the order (Fig. 5B).

4 The urohyal is the primitive site of origin for the *rectus communis* in ctenosquamates [28], [36]. Lophiiforms lack a urohyal [62] and have a *rectus communis* originating from the dorsal hypohyal. In *Ogcocephalus*, the origin of the *rectus communis* involves both the dorsal hypohyal and the anteromedial region of the anterior ceratohyal.

### Blenniiformes

Examined blenniiforms share the following modification in the infrabranchial musculature.

**Figure 8:**
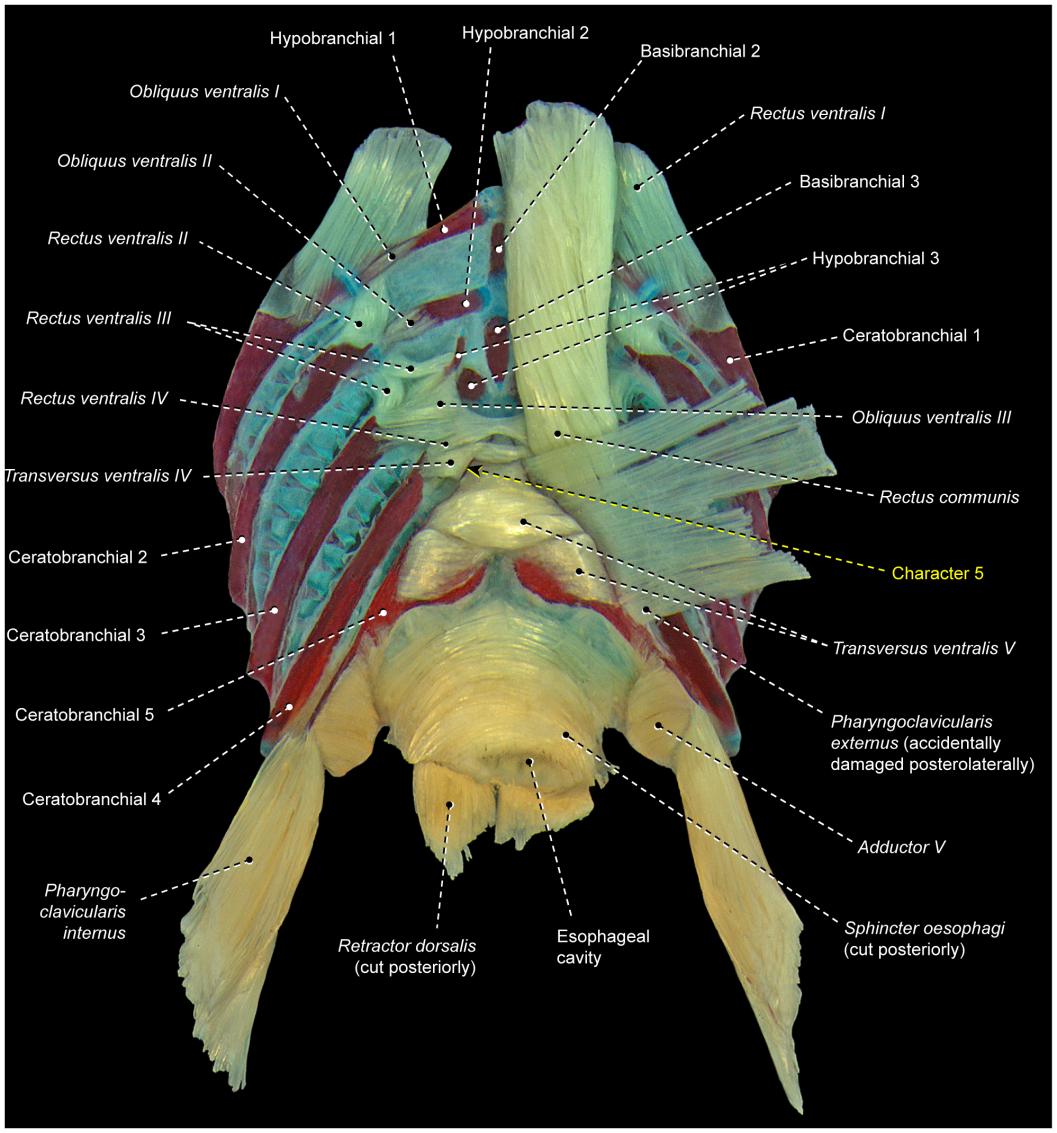
Branchial musculoskeletal system of *Scartella cristata* (Blenniiformes: Blenniidae). MZUSP 60573. Ventral view; right *pharyngoclavicularis externus* and *rectus communis* removed.

**Figure 9:**
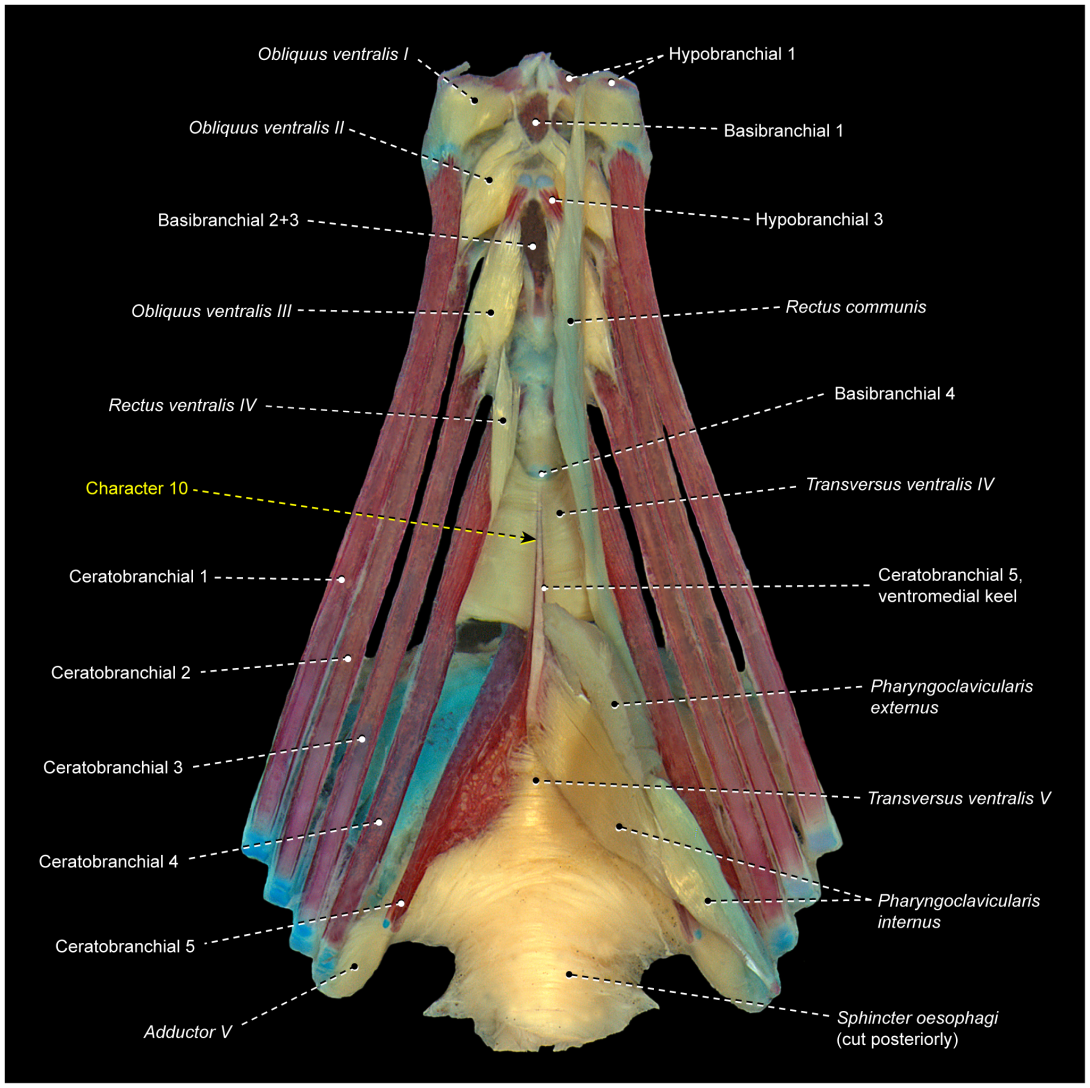
Branchial musculoskeletal system of *Cichla* cf. *piquiti* (Labriformes: Cichlidae). LIRP 6317. Ventral view; gill rakers and right *pharyngoclavicularis externus*, *pharyngoclavicularis internus*, and *rectus communis* removed.

5 Primitively in percomorphs, *transversus ventralis IV* is an unpaired muscle that connects ceratobranchials 4 of each side (Figs. 3, 4, 7). In *Enneanectes* and *Scartella* the anteroventral portion of *transversus ventralis IV* retains these primitive attachments, whereas the posterodorsal portion of the muscle attaches to the anterolateral margin of ceratobranchial 5 (Fig. 8). This condition is also present in other blenniiform families [10] and was considered a synapomorphy for the order by Springer and Orrel ([64]: character 35, state 1). Wiley and Johnson [1] incorrectly listed the absence of *transversus ventralis IV* ( = character 35, state 2 of Springer and Orrel [64]) as a synapomorphy for the Blenniiformes. Among all other examined percomorphs, only exocoetoids and labriforms exhibit a condition similar, but not identical, to that in blenniiforms. In exocoetoids and labriforms the dorsal portion or the entire *transversus ventralis IV* inserts on a conspicuous ventromedial keel of ceratobranchial 5, rather than on the anterolateral margin of ceratobranchial 5 (Fig. 9; see character 10 below).

### Callionymoidei and Gobiesocoidei

Some traditional classifications considered gobiesocoids as closely related to batrachoidiforms and lophiiforms [47], [53], [65]. That hypothesis was later contested by other morphological studies that presented evidence supporting the grouping of Gobiesocoidei (Gobiesocidae) with Callionymoidei (Draconettidae+Callionymidae; Fig. 5C) [1], [44], [66]–[68]. Molecular phylogenies, on the other hand, recovered gobiesocoids, callionymoids, batrachoidiforms, and lophiiforms as not closely related to each other [4], [5], [8], [9], [69]. The following evidence from the infrabranchial myology supports a gobiesocoid-callionymoid alignment.

**Figure 10:**
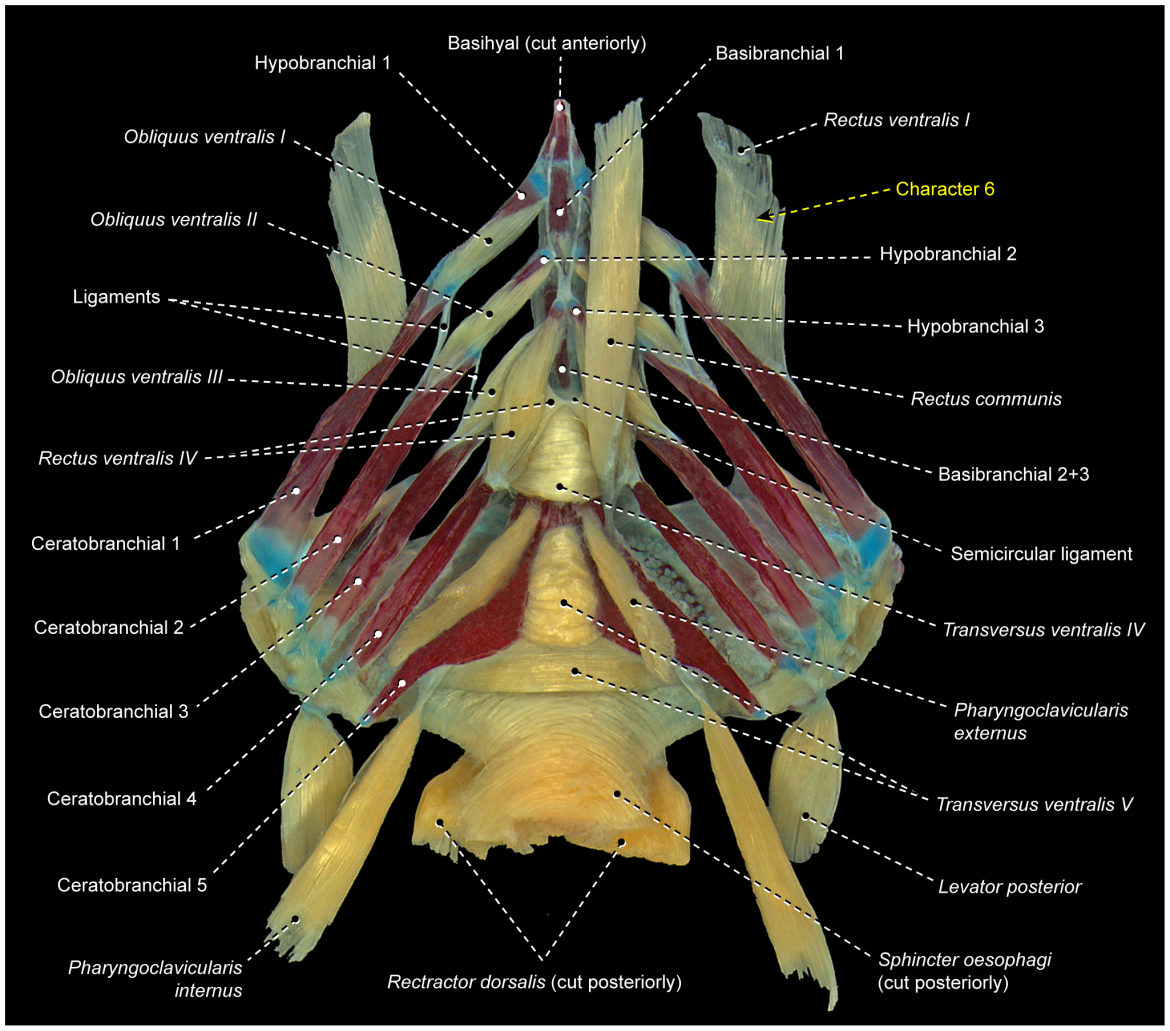
Branchial musculoskeletal system of *Synchiropus agassizii* (Gobiesociformes: Callionymidae). MZUSP 66707. Ventral view; gill rakers and right *rectus communis* removed.

6 *Rectus ventralis I* is a muscle apparently derived from an anterolateral subdivision and/or expansion of *obliquus ventralis I* that acquires a novel attachment on the hyoid arch, usually the dorsal hypohyal (Fig. 8) [12]. In gobiesocoids and callionymoids, *rectus ventralis I* is a flat muscle anterolaterally oriented toward its origin and with a broad insertion solely on the anterolateral border of the ossified portion of ceratobranchial 1 (Fig. 10) [70]. *Rectus ventralis I* is a conspicuous muscle present on both sides of the examined gobiesocoid and callionymid, but it is very small and present only on the right side of the examined draconettid. All other known percomorphs either lack *rectus ventralis I* (Figs. 3, 6, 7, 9) or have this muscle anteromedially oriented towards its origin (Fig. 8) and usually with a narrow insertion on hypobranchial 1 and/or the anterior cartilaginous tip of ceratobranchial 1. The derived condition of *rectus ventralis I* of gobiesocoids and callionymoids thus supports the hypothesis that these taxa are sister groups (Fig. 5C) [1], [44], [66]–[68].

The examined gobiesocoid, *Gobiesox strumosus*, possesses *recti ventrales II* and *III*, which likely represent anterolateral subdivisions of *obliqui ventrales II* and *III*, respectively. The draconettid *Centrodraco oregonus* also has *rectus ventralis II*. Dietz [70] reported the presence of *rectus ventralis II* in *Callionymus lyra*, but this muscle is absent in the callionymid herein examined (Fig. 10). Some, but not all, members of the Blenniiformes also have *recti ventrales II* and *III* (Fig. 8). Because several independent studies proposed that blenniiforms and gobiesociforms form a monophyletic group [4], [7], [55], [64], the precise optimization of the presence of *recti ventrales II* and *III* in these taxa is ambiguous. To further complicate the issue, other apparently non-closely related percomorphs also have *recti ventrales II* and/or *III*: some acanthuriforms [71], [72], tetraodontiforms [73], cottoids [74], [75], and gasterosteiforms [25].

### Gobiiformes

Monophyly of the Gobiiformes is strongly supported by both osteological [76]–[78] and molecular data [79], [80]. We identify the first putative myological synapomorphy for the order (Fig. 5D).

**Figure 11:**
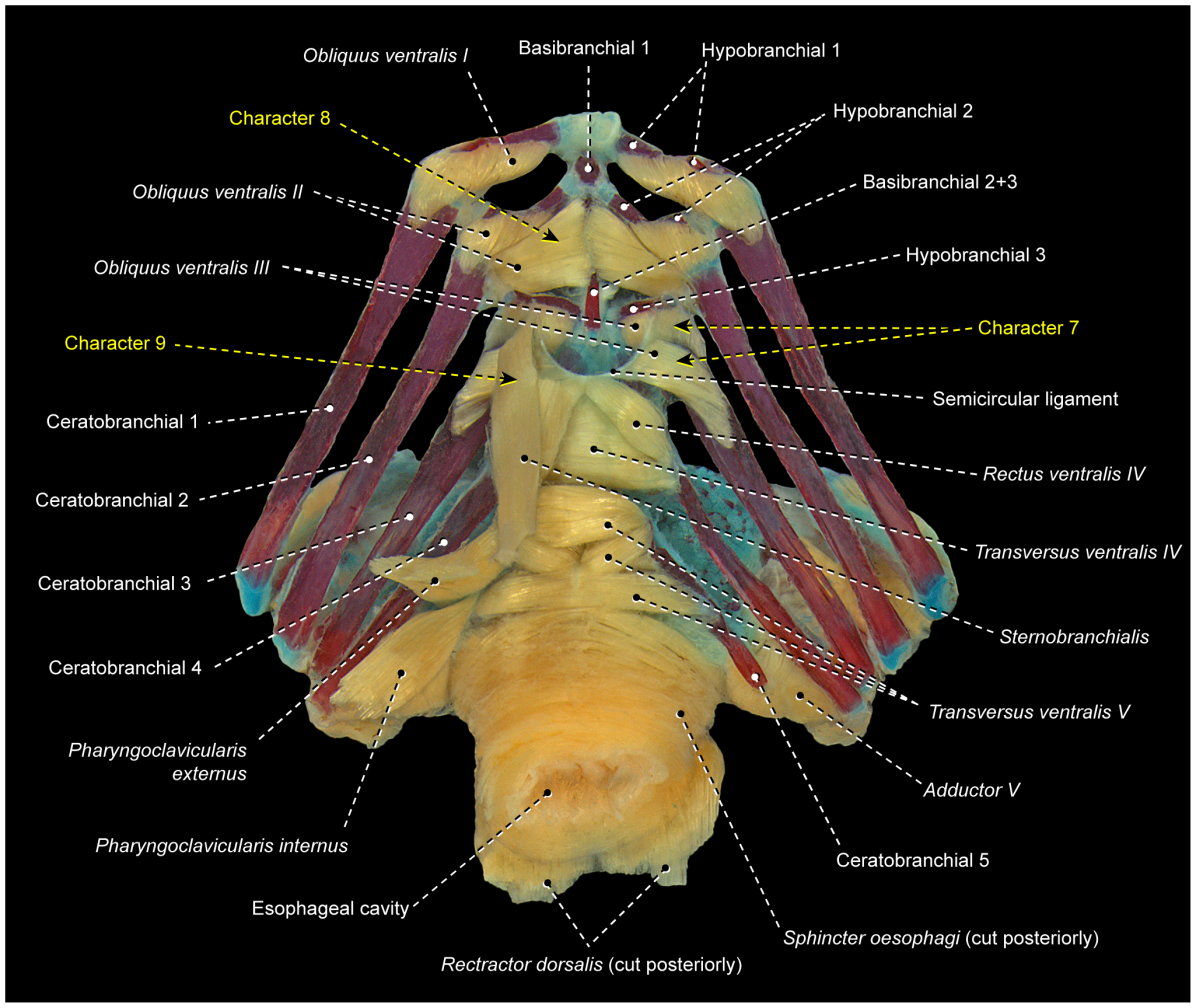
Branchial musculoskeletal system of *Bathygobius soporator* (Gobiiformes: Gobiidae). MZUSP 66368. Ventral view; gill rakers, *rectus communis*, and left *pharyngoclavicularis externus*, *pharyngoclavicularis internus*, and *sternobranchialis* removed.

7 Examined gobiiforms (including the putatively basal Rhyacichthyidae) possess *obliquus ventralis III* divided into an anterodorsal section that directly arises from ceratobranchial 3 and a posteroventral section that originates from the semicircular ligament (Fig. 11). Contrarily, most acanthomorphs, including taxa commonly suggested as possible gobiiform closest relatives [68], [79], [80], have undivided *obliquus ventralis III* (Figs. 3, 4, 6, 9, 10). Among other examined acanthopterygians, a similarly divided *obliquus ventralis III* was found only in the batrachoidiforms *Batrachoides* and *Porichthys*. In view of the long phylogenetic distance between batrachoidiforms on the one hand and gobiiforms on the other, parsimony considerations indicate that the divided *obliquus ventralis III* is convergent in the two orders (Fig. 5B, D).

Prior anatomical studies placed the Rhyacichthyidae and Odontobutidae as successive sister groups of the clade formed by all remaining gobiiforms [76], [77]. Molecular phylogenies contrastingly grouped these families into a monophyletic lineage that is, in turn, sister to remaining gobiiforms [79], [80]. The following newly discovered characters corroborate the former hypothesis advanced by morphologists (Fig. 5D).

**Figure 12:**
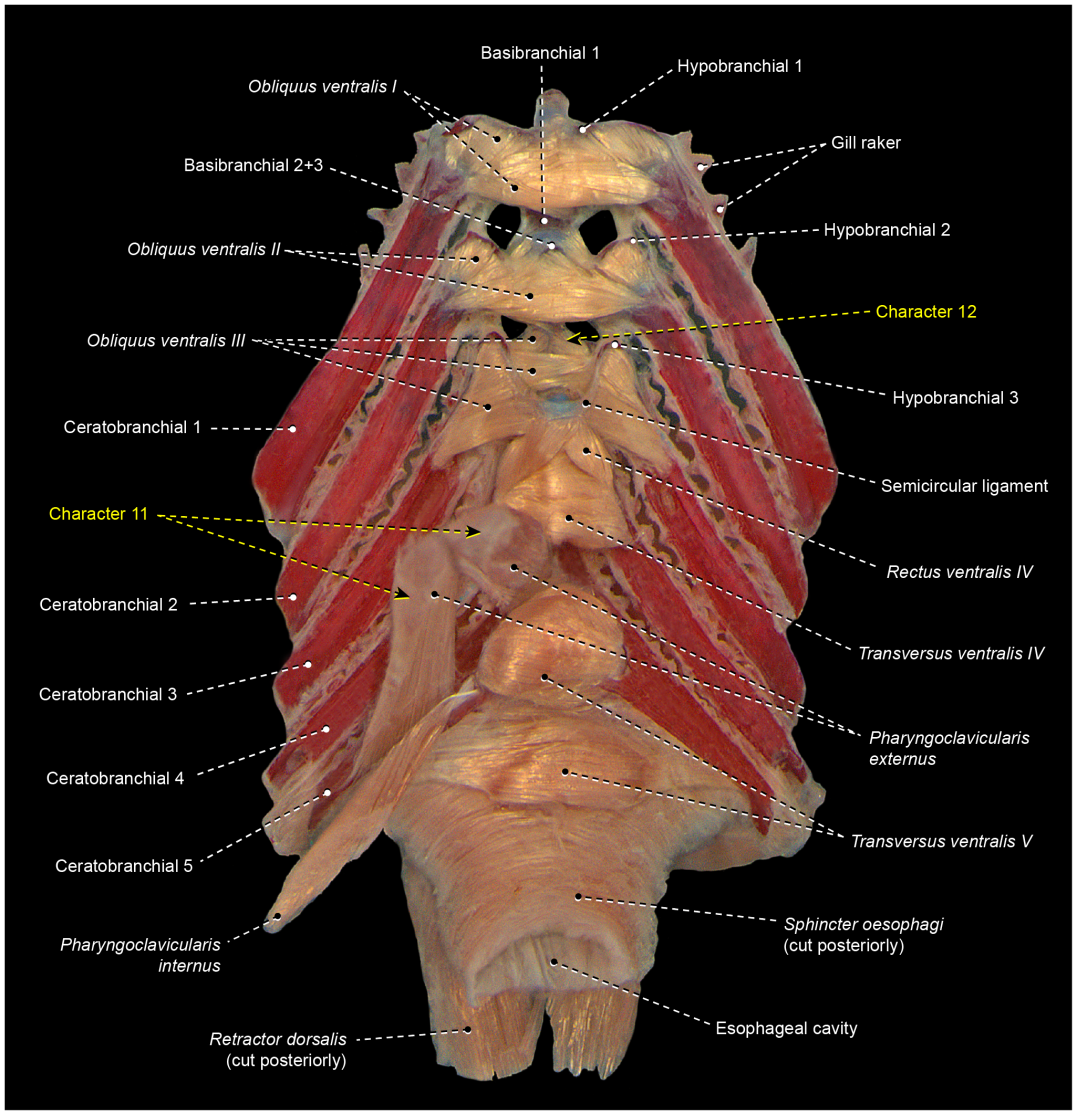
Branchial musculoskeletal system of *Fundulus heteroclitus* (Cyprinodontiformes: Fundulidae). MZUSP 67017. Ventral view; *rectus communis* and left *pharyngoclaviculares externus* and *internus* removed.

**Figure 13:**
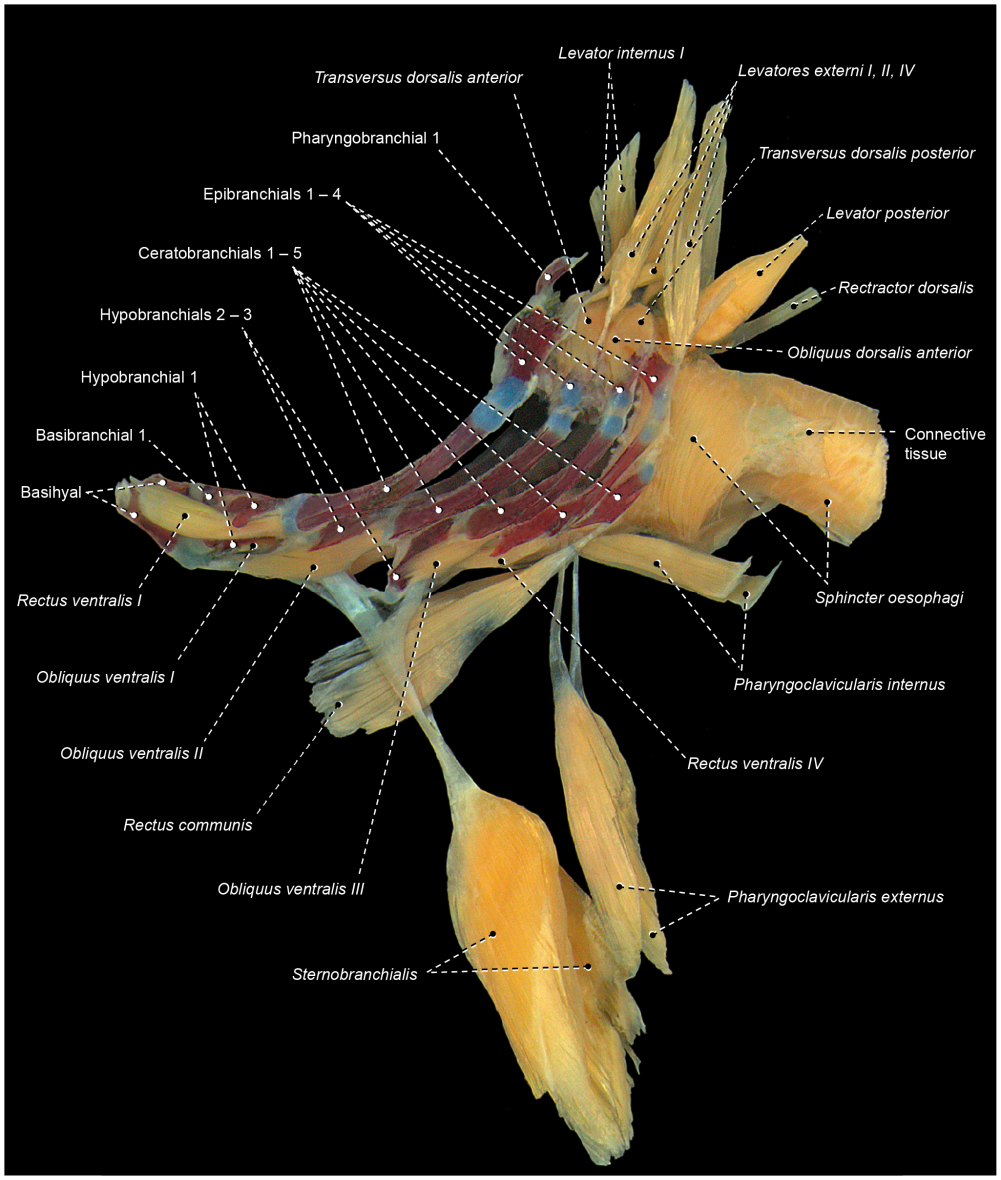
Branchial musculoskeletal system of *Antigonia capros* (Caproiformes: Caproidae). MZUSP 108164. Left lateral view; gill rakers removed. Anterior portion of basihyal accidentally cut.

8 Percomorphs primitively have an undivided *obliquus ventralis II* that runs from ceratobranchial 2 to hypobranchial 2 (Figs. 3, 6, 7, 9, 10). In all gobiiforms except rhyacichthyids, *obliquus ventralis II* is completely subdivided into an anterolateral section, which retains the plesiomorphic muscle attachments, and a posteromedial section, which originates from basibranchial 2+3 ( = O'_2_ muscle of Dietz [70]; Fig. 11). Among all other known acanthopterygians, a similar but not identical condition is found only in the Atherinomorphae and Gasterosteiformes [25]. These taxa have an *obliquus ventralis II* originating from both hypobranchial 2 and basibranchial 2+3, but the muscle is never subdivided into two separate sections as it is in gobiiforms (Fig. 12). Furthermore, gobiiforms are apparently not closely related to either atherinomorphs or gasterosteiforms [7], [56], [67], [68], [80]–[82]. Consequently, the presence of a separate medial section of *obliquus ventralis II* originating from basibranchial 2+3 may be interpreted as an evidence for the monophyly of the non-rhyacichthyid gobiiforms (Fig. 5D).
9 Rhyacichthyids exhibit the primitive acanthopterygian condition in which the *sternohyoideus* originates from the cleithrum and inserts solely on the urohyal. In all remaining gobiiforms, a dorsomedial segment of the *sternohyoideus* attaches to the anteroventral tip of hypobranchial 3 (Fig. 11) [70]. This muscle segment is partially separated from the main bulk of the *sternohyoideus* in odontobutids and completely separated from the *sternohyoideus* in the remaining gobiiforms. Following Winterbottom [33], this muscle segment attached to hypobranchial 3 may be designated as *sternobranchialis* ( = ‘Ad’ of Dietz [70]). In *Dormitator*, the insertional tendon of the *sternobranchialis* further extends anteriorly to attach to both hypobranchial 2 and 3. In an ordered series of transformation, the attachment of the dorsomedial fibers of the *sternohyoideus* to hypobranchial 3 (that is, an at least partially differentiated *sternobranchialis*) may thus be taken as a synapomorphy for the non-rhyacichthyid gobiiforms (state 1), whereas a fully separated *sternobranchialis* is considered a synapomorphy for the clade formed by all gobiiforms except the Rhyacichthyidae and Odontobutidae (state 2; Fig. 5D). Among percomorphs, a *sternobranchialis* is also present in tetraodontiforms and the caproiform *Antigonia* (Fig. 13), taxa that are distantly allied to gobiiforms [7], [55], [56], [64], [68], [80], [82]. Additionally, the *sternobranchialis* in gobiiforms is markedly different from that of tetraodontiforms and caproiforms. The gobiiform *sternobranchialis* has a parallel arrangement of fibers along almost its entire extent, a nearly horizontal orientation, antimeres distant from each other, and a single undivided insertion on hypobranchial 3 or hypobranchials 2 and 3 (Fig. 11). In contrast, the *sternobranchialis* of caproiforms and tetraodontiforms is characterized by a bulged origin and strongly flattened insertion, a nearly vertical orientation, antimeres much closer to one another, and an insertional aponeurosis splitting into two or more tendons that attach to several bones of the branchial and/or hyoid arches (Fig. 13) [73]. Although such differences in themselves are not decisive refutation of homology, they provide further corroboration that the gobiiform *sternobranchialis* evolved independently (Fig. 5D) of that in caproiforms and tetraodontiforms (see character 29).

### Labriformes and Pholidichthyiformes

In contrast to the situation in most other percomorph groups, the branchial musculature of labriforms has been extensively studied [10], [19], [21], [70], [83]–[85]. The enigmatic *Pholidichthys*, sole genus of Pholidichthyiformes, has been indecisively aligned with different percomorph groups, including blenniiforms, gobiiforms, labriforms, and trachiniforms [19], [65], [86]–[88]. Stiassny and Jensen [19] highlighted that labriforms and pholidichthyiforms apparently share several similarities in the branchial skeleton, and the analysis of Springer and Orrel [64] placed *Pholidichthys* within the Labriformes. The present study confirms that pholidichthyiforms exhibit a putative labriform synapomorphy.

10 In the primitive condition for percomorphs, *transversus ventralis IV* attaches solely to the contralateral ceratobranchials 4, completely bypassing ceratobranchial 5 (Figs. 3, 4, 6, 7, 10–12). In labriforms and pholidichthyiforms, at least the dorsal portion of *transversus ventralis IV* attaches to a conspicuous ventromedial keel of ceratobranchial 5 (Fig. 9) [10], [19], [21], [70], [83]–[85]. The same condition is also present in exocoetoid beloniforms, a group well nested within the Atherinomorphae. On the grounds of parsimony, the *transversus ventralis IV* muscle attaching to the ventral keel of ceratobranchial 5 of exocoetids is interpreted as having independently evolved from that of labriforms [19] and pholidichthyiforms.

As noted above (character 5), blenniiforms have only the posterior portion of *transversus ventralis IV* attached to the anterolateral region of ceratobranchial 5 (Fig. 8). In our opinion, this condition is clearly distinct from that in both labriforms, pholidichthyiforms, and exocoetids in which the entire, or only the dorsal part, of the muscle attaches to a ventromedial keel of ceratobranchial 5 (Fig. 9). Nevertheless, Springer and Orrel [64] coded these different conditions under the same character state: *transversus ventralis IV* attached to ceratobranchial 5 (their character 35, state 1). Under that codification, the analysis of Springer and Orrel [64] did not recover the insertion of *transversus ventralis IV* on ceratobranchial 5 as a synapomorphy for their clade 5, which comprises the Labriformes plus Pholidichthyiformes.

Kaufman and Liem [89] and Stiassny and Jensen [19] listed a second myological synapomorphy for the Labriformes: the *sphincter oesophagi* lacking a dorsal subdivision. The referred dorsal subdivision of the *sphincter oesophagi* corresponds to the “*sphincter oesophagi* division” or SOD muscle of Springer and Johnson [10], which passes dorsal to the insertion of the *retractor dorsalis*. Although the *sphincter oesophagi* was included in the present analysis, it seems that the unequivocal identification of SOD in some taxa necessarily requires the study of other dorsal branchial muscles, especially the *transversus dorsalis posterior*. The latter muscle occupies a position similar to that of SOD and those two muscles are sometimes continuous with each other ([10]: p. 13). As the dorsal branchial muscles were not investigated in the present study, we could not positively determine the presence or absence of the SOD in examined percomorphs and must leave this question unresolved.

The monophyly of the Labriformes, with the possible inclusion of *Pholidichthys* [64], is currently supported only by characters from the branchial arches [1], [19], [64], [89], and the lack of evidence from other anatomical systems is often cited as a major criticism of the supposed monophyly of the order [1], [90]. All molecular analyses to date have recovered a polyphyletic Labriformes but, interestingly, *Pholidichthys* is often resolved as sister to the labriform family Cichlidae [8], [9], [56], [91]. Resolution of these conflicts is beyond the scope of the present study.

### Mugiliformes and Atherinomorphae

Stiassny [81] proposed mugiliforms and atherinomorphs as sister taxa (Fig. 5E) on the basis of a number of shared morphological specializations, including the following character of the infrabranchial musculature.

11 In the primitive percomorph condition, the *pharyngoclavicularis externus* is an undivided muscle that inserts on the anteroventral region of ceratobranchial 5 (Figs. 3, 6, 7, 8, 9, 10, 11, 13). In mugiliforms and non-exocoetoid atherinomorphs, this muscle is dorsally divided into an anterior and a posterior section, which insert on the anteroventral and posteroventral regions of ceratobranchial 5, respectively (Fig. 12). The present study confirms the distribution and putative validity of this character as a synapomorphy for the clade Mugiliformes+Atherinomorphae (Fig. 5E). Exocoetoid beloniforms have a single undivided *pharyngoclavicularis externus* inserting solely on the posteroventral region of ceratobranchial 5, thus resembling the primitive state for the Percomorphacea. Stiassny ([24]: p. 6) interpreted the exocoetoid condition as the result of a loss of the anterior subdivision of the *pharyngoclavicularis externus*, rather than a reversal to the ancestral percomorph state. We have not disclosed new evidence either for or against such interpretation.

Stiassny [24] proposed a second character of the infrabranchial musculature as supporting a mugiliform-atherinomorph relationship: the “*pharyngohyoideus* muscle [ = *rectus communis*] reduced to a small fan-shaped muscle with an elongate tendon”. This character was later discarded by Stiassny [81] because of the extensive degree of variation detected among a broader range of percomorphs, which renders the delimitation of discrete character-states problematic. Such observations and conclusions are supported by the present study.

The monophyly of the Atherinomorphae is almost unanimously accepted by phylogenetic analyses based on both genetic and phenotypic data sets (Fig. 5E) [7]–[9], [24], [56], [64], [92]–[96]. The following derived condition was identified in the infrabranchial musculature of the examined atherinomorphs.

12 Percomorphs primitively have an undivided *obliquus ventralis III* originating from the ventral face of hypobranchial 3 and inserting on the ventrolateral region of ceratobranchial 3 (Figs. 3, 6–7, 9, 10, 13). In four of the five examined atherinomorphs, *obliquus ventralis III* is expanded dorsomedially in such a way that the insertion of the muscle involves ceratobranchial 3 and the dorsal face of hypobranchial 3, whereas its origin extends over basibranchial 2+3 and the ventral face of hypobranchial 3 (Fig. 12). This condition was not found elsewhere among percomorphs, and is thus hypothesized to be synapomorphic for the Atherinomorphae (Fig. 5E), with a reversal in the cyprinodontiform *Hypsolebias*, which exhibits the primitive percomorph condition.

### Scombroidei

The Scombroidei has long been recognized as a natural group by morphologists [44], [65], [97]–[103], though the exact composition of the suborder has varied over time. In the morphological analysis of Johnson [98], the Scombroidei includes the Sphyraenidae and excludes the monotypic Scombrolabracidae and Pomatomidae, but the two later families are recovered as the successive sister groups of the suborder (Fig. 5F). Molecular analyses, however, resolve the Scombroidei as polyphyletic [4], [5], [8], [9], [69], [104]. The following unique apomorphic condition of the infrabranchial musculature supports the monophyly of the Scombroidei *sensu* Johnson [98].

**Figure 14:**
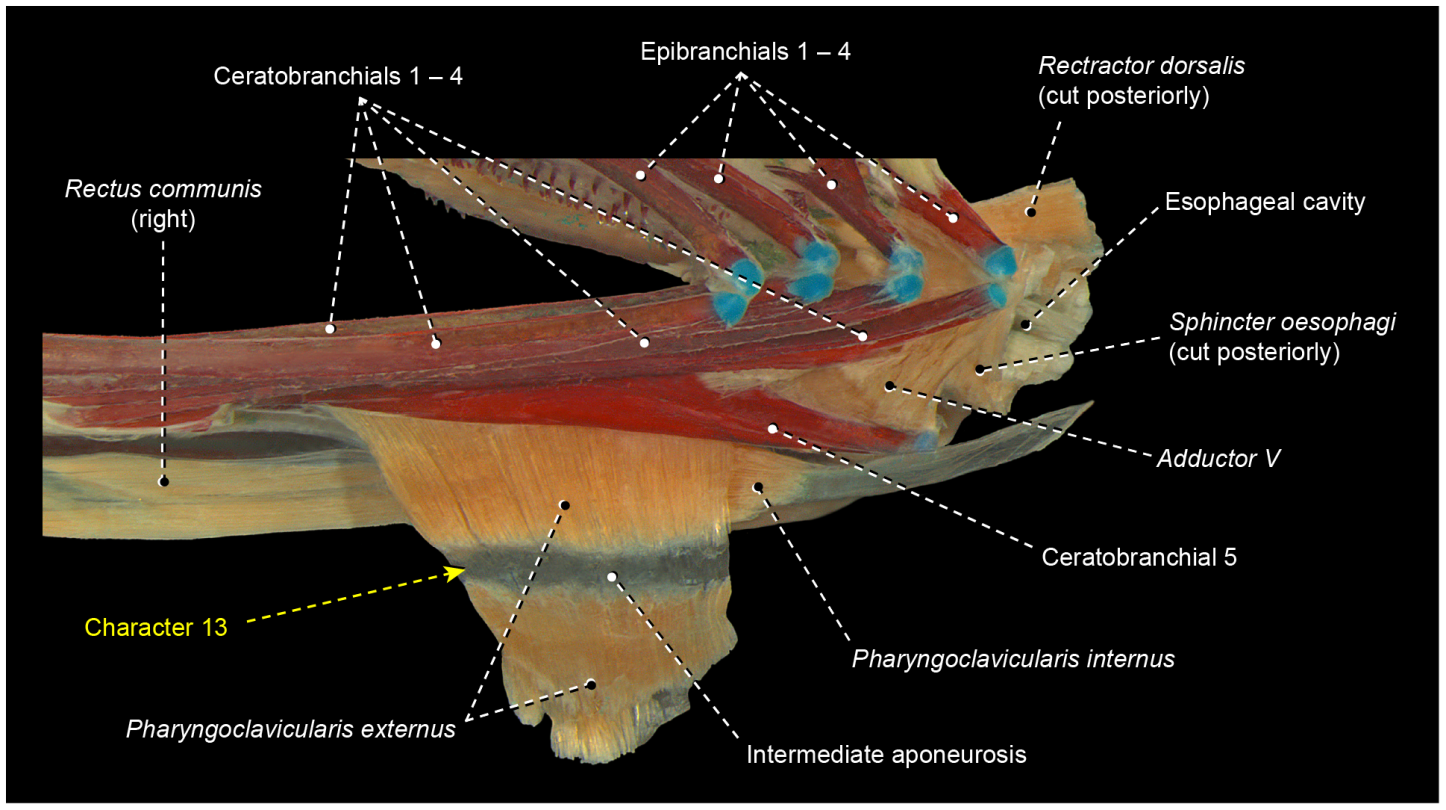
Ventroposterior region of branchial musculoskeletal system of *Sphyraena obtusata* (Scombriformes: Sphyraenidae). MZUSP 37378. Left lateral view; gill rakers and left *rectus communis* removed.

13 In most percomorphs, including *Scombrolabrax* and *Pomatomus*, the *pharyngoclavicularis externus* lacks any intermediate aponeurosis ( = a laminar, flattened tendon; Figs. 3, 6, 7, 8, 9, 10, 11, 12, 13). Most scombroids contrastingly have an intermediate aponeurosis at the mid-length of this muscle (Fig. 14). This aponeurosis runs across the entire anteroposterior expanse of the *pharyngoclavicularis externus* in sphyraenids and the scombrid *Scomberomorus* (M. Nakae, pers. comm.), but only at the anterior portion of the same muscle in gempylids, xiphiids, and the scombrids *Euthynnus*, *Scomber*, and *Thunnus* (M. Nakae, pers. comm.). Istiophorids exhibit a further modified condition in which a broad aponeurosis is located at the dorsal end (insertion) of the *pharyngoclavicularis externus*. Trichiurids, in turn, are the only investigated scombriforms lacking an aponeurosis in that muscle ([105], pers. obs.). Among all other examined acanthopterygians, only *Kyphosus* also possesses an intermediate aponeurosis at the anterior region of the *pharyngoclavicularis externus*. As both morphological and molecular evidence strongly indicate that *Kyphosus* and scombroids are more closely related to other examined percomorphs than to each other [104], [106]–[108], the derived conditions of the *pharyngoclavicularis externus* in each taxon are most parsimoniously considered as convergent. Therefore, the presence of an intermediate aponeurosis on the *pharyngoclavicularis externus* may constitute an additional synapomorphy for the Scombroidei *sensu* Johnson [98], with a reversal in Trichiuridae and a secondary change in the position of the aponeurosis occurring in Istiophoridae (Fig. 5F).

Mugiliforms and most atherinomorphs have a *pharyngoclavicularis externus* divided into an anterior and a posterior section (Fig. 12; see character 11). In *Mugil* only the anterior section of this muscle has an intermediate aponeurosis. This condition is thus not homologous to that of scombroids.

### Stromateiformes

**Figure 15:**
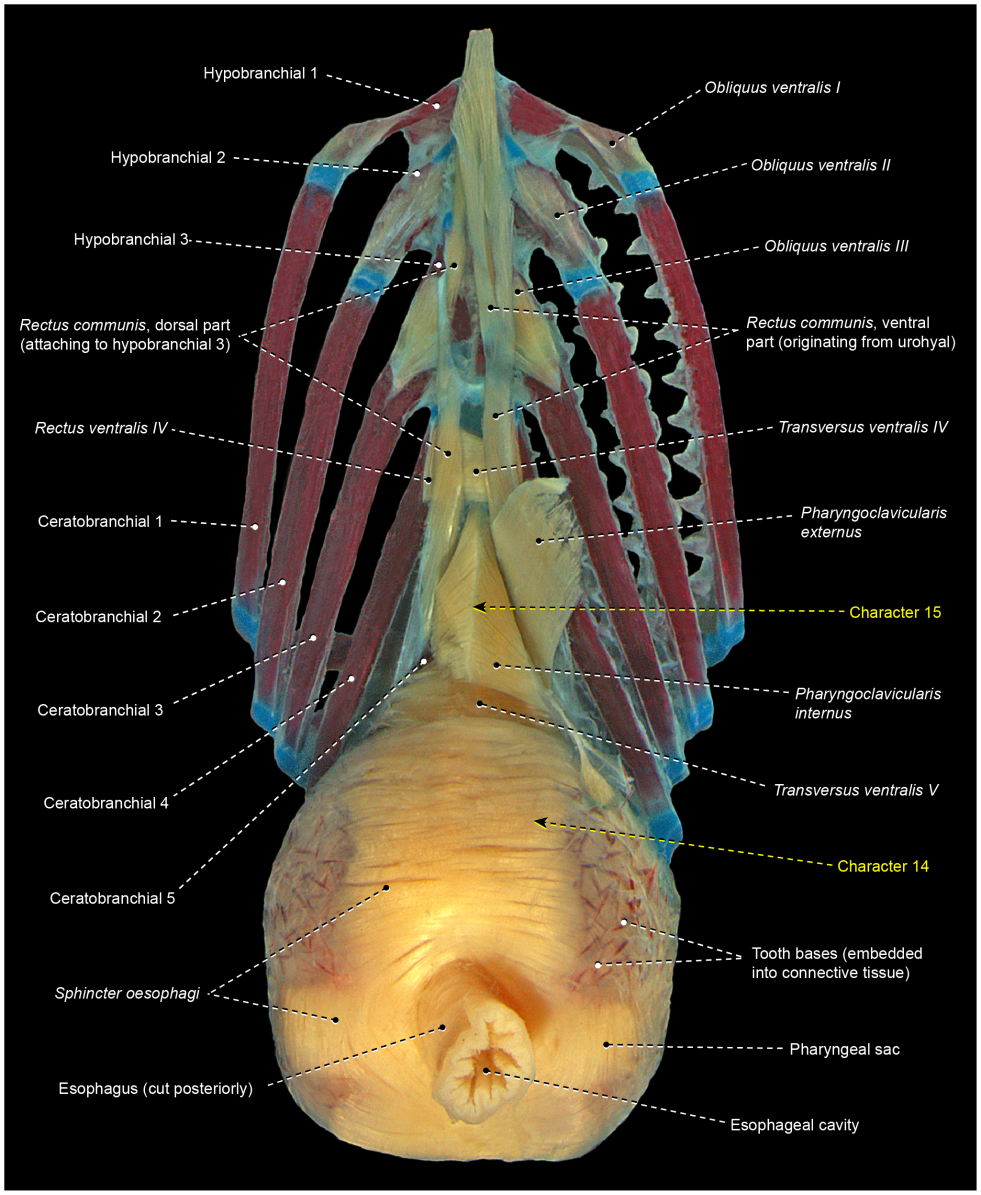
Branchial musculoskeletal system of *Peprilus triacanthus* (Stromateiformes: Stromateidae). MZUSP 112356. Ventral view; right gill rakers, ventral part of the *rectus communis*, and *pharyngoclavicularis externus* removed; right *pharyngoclavicularis internus* cross-sectioned.

For more than a century anatomists have recognized the Stromateiformes as a natural group, mainly because of their common possession of a toothed saccular outgrowth located just posterior to the last branchial arch (Fig. 15) [100], [109]–[112]. Such a structure, called a pharyngeal sac, is unparalleled within the Actinopterygii and constitutes the most notable anatomical feature of stromateiforms. *Amarsipus carlsbergi* lacks a pharyngeal sac, but Haedrich [110] included this species within the Stromateiformes (Fig. 5G) because of other supposedly apomorphic conditions shared with remaining stromateiforms. The alignment of amarsipids with the other stromateiforms has been questioned with the demonstration that all but one character used to group these taxa have wider distributions among percomorphs [1], [10]. Molecular phylogenies, strangely, split non-amarsipid stromateiforms into several separate clades that appear intercalated with bramids, caristiids, chiasmodontids, icosteids, and several scombroid lineages (Scombroidei is also resolved as polyphyletic) [8], [9], [108].

14 We confirm the presence of a toothed pharyngeal sac as a remarkable specialization unique to non-amarsipid stromateiforms (Fig. 15). This sac is covered externally by a greatly expanded *sphincter oesophagi*, which presents several differentiated fiber bundles. The contraction of this specialized muscle provides the primary force for the food processing that takes place in the pharyngeal sac. In anatomical descriptions and illustrations of Springer and Johnson [10], the stromateiform pharyngeal sac is referred to as an “epibranchial organ” [EO]. Depending on the phylogenetic allocation of the Amarsipidae in the morphology-based phylogenies of the Stromateiformes, the presence of a pharyngeal sac and its associated specialized *sphincter oesophagi* is optimized as either a synapomorphy for the clade formed by all non-amarsipid stromateiforms [111] (Fig. 5G) or a synapomorphy for the whole order, with a secondary reversal in *Amarsipus* [113]. All molecular analyses including a broader taxonomic sampling of stromateiforms and their possible closer relatives [9], [108] yielded topologies that necessarily lead to the unlikely hypothesis that the pharyngeal sac and its associated dental and muscular specializations evolved three times independently. To date, *Amarsipus* has not been included in any molecular phylogeny.
15 The bramid *Brama* and the non-amarsipid stromateiforms have a *pharyngoclavicularis internus* inserting onto a sagittal raphe shared with its antimere (Fig. 15). This raphe is dorsally continuous with the sagittal raphe of *transversus ventralis V*, which extends anteriorly to attach to the anterior tip of ceratobranchial 5. An insertional raphe for the *pharyngoclavicularis internus* was not found elsewhere among the examined acanthopterygians (Figs. 3, 6, 7, 8, 9, 10, 11, 12). Though molecular analyses allocated bramids and stromateiforms broadly into the same large monophyletic lineage [8], [9], [108], those two taxa were hypothesized as more closely aligned to other percomorphs lacking a sagittal raphe for the *pharyngoclavicularis internus*. If such scheme is accepted, then bramids and stromateiforms independently evolved this raphe, which thus might be interpreted as additional evidence for the monophyly of the non-amarsipid stromateiforms (Fig. 5G) [110], [111].

### Modifications of uncertain systematic value

Some characters of the infrabranchial musculature apparently exhibit a mosaic distribution that is not immediately compatible with any known previous hypothesis of phylogenetic relationships among the major percomorph groups. These characters should not be seen as phylogenetically uncorrelated in an absolute sense, or as “noise” of lesser comparative relevance. In any morphological complex, different characters exhibit different degrees of homoplasy and this also applies to the infrabranchial musculature. The characters in this section may be diagnostic to less inclusive groups of percomorphs than those discussed in the preceding sections and/or indicate the existence of clades that have not been previously proposed in the literature. However, a correct interpretation of the phylogenetic significance of these characters will require broader phylogenetic analyses incorporating both additional taxa and characters from other body systems. In order to make these data available for future studies, characters of the infrabranchial musculature with undetermined phylogenetic signal are listed below.

16 Percomorphs primitively lack *rectus ventralis I* (Figs. 3, 6, 7, 9, 10, 11, 12, 14, 15). Among examined taxa, *rectus ventralis I* is present in *Abudefduf, Acanthurus, Anabas, Antigonia* (Fig. 13), *Badis, Capros, Centrodraco, Centropyge, Chaetodon, Dactylopterus, Drepane, Elassoma, Gobiesox, Luvarus, Mastacembelus, Parachanna, Pholidichthys, Scartella* (Fig. 8), *Stephanolepis, Synchiropus* (Fig. 10), *Thalassoma, and Triacanthus.*
17 In the plesiomorphic condition for percomorphs, *obliquus ventralis I* originates solely from hypobranchial 1 (Figs. 3, 6, 7, 8, 9, 10, 11). In *Exocoetus, Fundulus* (Fig. 12), *Gasterosteus, Odontobutis, Parexocoetus*, and *Pungitius* the origin of that muscle involves both hypobranchial 1 and basibranchial 1.
18 The batrachoidiforms *Thalassophryne* and *Triathalassothia* lack *obliquus ventralis II*. In *Antennarius, Batrachoides* and *Lophius* this muscle is vestigial. All remaining acanthopterygians exhibit a well-developed *obliquus ventralis II* muscle (Figs. 3, 4, 6, 7, 8, 9, 10, 11, 12, 15).
19 In the primitive percomorph condition, *obliquus ventralis II* is an undivided muscle that arises solely from hypobranchial 2 and inserts on ceratobranchial 2 (Figs. 3, 7, 8, 9, 10, 15). Atherinomorphs, gasterosteiforms [25], and the labrid *Thalassoma* have this undivided muscle expanded medially at its origin, that includes both hypobranchial 2 and basibranchial 2+3 (Fig. 12). The same two bones also serve as origin for *obliquus ventralis II* in gobiiforms. However, in gobiiforms the muscle is completely subdivided into an anterolateral section that originates solely from hypobranchial 2, and a posteromedial section, which arises solely from basibranchial 2+3 (character 8; Fig. 11).
20 Percomorphs primitively have *obliquus ventralis II* originating from hypobranchial 2 only (Figs. 3, 7, 8, 9, 10, 15). In *Antigonia*, one of the two examined specimens of Capros, triacanthids, balistoids, drepaneids, ephippidids, scatophagids, most acanthuriforms (except *Luvarus* and *Siganus*), chaetodontids, pomacentrids, and pristolepidids, *obliquus ventralis II* has an anterior projection that attaches to the sagittal elements of the anteriormost pharyngeal arches (urohyal, basihyal, or basibranchial 1) (Fig. 13). Some authors designate this anterior projection as *rectus ventralis II* [71]–[73], but in our opinion such a designation might be confusing in taxa having this muscle projection completely continuous with the remainder of *obliquus ventralis II*. A so-called *rectus ventralis II* is also present in a few internested subgroups within the Blenniiformes (Fig. 8), Gasterosteiformes [25], and Gobiesociformes. Nevertheless, in these three orders, *rectus ventralis II* alternatively inserts on the paired ventrolateral elements of the first branchial arch (hypobranchial 1 and/or ceratobranchial 1).
21 *Transversus ventralis IV* may exhibit a sagittal raphe either along its entire anteroposterior extent (batrachoidiforms, zeiforms, *Stephanolepis*, and *Triacanthus*; Fig. 7) or the posterior portion of the muscle only *(Apogon, Ctenopoma)*. In most examined taxa, however, *transversus ventralis IV* completely lacks a sagittal raphe (Figs. 3, 4, 6, 7, 8, 10–12, 15).
22 In most examined acanthopterygians *transversus ventralis V* has a sagittal raphe across its entire anteroposterior extent (Figs. 3, 15). *Amniataba, Bathygobius* (Fig. 11), *Batrachoides, Bembrops, Centropomus, Chaetodon, Cynoscion, Graus, Dormitator, Lutjanus, Nototheniops, Odontobutis, Parachanna, Parexocoetus, Rhyacichthys, Scartella* (Fig. 8), *Scorpis, Thalassophryne*, and *Triathalassothia* have a sagittal raphe restricted to only the posterior portion of the muscle. *Transversus ventralis V* completely lacks a sagittal raphe in *Acanthurus, Anabas, Antennarius, Astroscopus, Atherinella, Badis, Centrodraco, Centropyge, Chaunax, Cichla* (Fig. 9), *Ctenopoma, Dactylopterus, Dules, Elassoma, Enneanectes, Gasterosteus, Girella, Gobiesox, Fundulus* (Fig. 12), *Hypsolebias, Kyphosus, Lophius, Lycodes, Microcanthus, Nandus, Ogcocephalus, Porichthys* (Fig. 7), *Pungitius, Scatophagus, Siganus, and Synchiropus* (Fig. 10).
23 Most examined acanthopterygians have the *sphincter oesophagi* and *transversus ventralis V* completely separated from each other (Figs. 3, 6–8, 10, 11, 12, 15). These muscles are partially continuous with each other in *Chaetodipterus, Cichla* (Fig. 9), *Drepane, Dules, Elassoma, Enneanectes, Gasterosteus, Lophius, Lycodes, Mastacembelus, Nototheniops, Ogcocephalus, Parachanna, Parexocoetus, Rhyacichthys, Synbranchus, and Triacanthus.*
24 Primitively in percomorphs, *transversus ventralis V* is undifferentiated into subsections (Figs. 3, 7, 9, 15). This muscle is differentiated in an anterior and a posterior section in *Anabas, Antennarius, Badis, Bathygobius* (Fig. 11), *Centropomus, Ctenopoma, Cynoscion, Dormitator, Enneanectes, Fundulus* (Fig. 12), *Hypsolebias, Lutjanus, Monodactylus, Nandus, Nototheniops, Odontobutis, Orthopristis, Pristolepis* (Fig. 6), *Scartella* (Fig. 8), *Stephanolepis, and Synchiropus* (Fig. 10).
25 Lower euteleosts apparently have the rectus communis originating from hypobranchial 3 only [28], [36]. Stiassny [36] and Lauder [28] considered the shift in origin of this muscle to the urohyal as a ctenosquamate synapomorphy. The vast majority of the examined acanthomorphs indeed have a *rectus communis* originating solely from the urohyal. In *Brama, Holocentrus, Peprilus* (Fig. 15), *Prionotus*, and *Psettodes* the ventral portion of the muscle arises from the urohyal, but a dorsal set of fibers attach to hypobranchial 3. However, *Paralichthys, Poromitra, Trichiurus, Zenion, and Zenopsis* have a *rectus communis* originating from hypobranchial 3 only, such as in the putative plesiomorphic condition for the Euteleosteomorpha. Lophiiforms uniquely have a *rectus communis* arising from the dorsal hypohyal (character 4).
26 The lophiiforms *Antennarius, Lophius*, and *Ogcocephalus* and the batrachoidiforms *Batrachoides* and *Thalassophryne* have the *rectus communis* inserting musculously on ceratobranchial 5. In all other examined acanthopterygians, this insertion is completely tendinous (Figs. 3, 6, 7, 10, 13, 15).
27 Most examined acanthopterygians have the insertion of the *pharyngoclavicularis internus* positioned anteromedial to the insertion of the *pharyngoclavicularis externus* (Figs. 3, 9, 11, 12, 14, 15). As a consequence, the former muscle passes medial to the latter. In examined anabantomorphs (Fig. 6), batrachoidiforms (Fig. 7), lophiiforms, uranoscopids, blenniiforms (Fig. 8), callionymoids (Fig. 10), gobiesocoids, mastacembeloids, synbranchoids, elassomatiforms, *Odontobutis,* and *Siganus*, the insertion of the *pharyngoclavicularis internus* is posterior to the insertion of the *pharyngoclavicularis externus. Dormitator, Gasterosteus,* and *Lycodes* alternatively have a *pharyngoclavicularis internus* inserting posterolateral to the *pharyngoclavicularis externus.*
28 In the primitive percomorph condition, the *pharyngoclavicularis externus* inserts musculously on ceratobranchial 5 (Figs. 3, 6, 7, 8, 9, 10, 11, 12, 14, 15). In *Antigonia, acanthurids,* and most tetraodontiforms, the insertion of this muscle is entirely mediated by tendon (Fig. 13) [71]–[73].
29 The caproiform *Antigonia* and non-tetraodontoid tetraodontiforms have a fully differentiated *sternobranchialis,* a muscle derived from the separation of the dorsomedial portion of the *sternohyoideus* (Fig. 13) [33]. Elsewhere in percomorphs, only gobiiforms also have a well-differentiated *sternobranchialis* (Fig. 11). However, the gobiiform *sternobranchialis* differs from that of *Antigonia* and tetraodontiforms in many features (see character 9) and analyses of different data sets (osteology, myology, and DNA) indicate that gobiiforms are not closely related to caproiforms or tetraodontiforms [7], [55], [56], [64], [68], [80], [82]. Therefore, the sternobranchialis of gobiiforms apparently is not homologous to the *sternobranchialis* of *Antigonia* and tetraodontiforms, which is characterized by having a bulged origin and strongly flattened insertion, a nearly vertical orientation, antimeres much closer to one another, and an insertional aponeurosis splitting into two or more tendons that attach to several bones of the branchial and/or hyoid arches (Fig. 13).

## Concluding Remarks

The infrabranchial musculature has been unexplored in most fish groups, and the present study shows that many modifications in this system may have important implications to the higher-level phylogeny of percomorphs. Data from the infrabranchial muscles notably support several hypotheses of percomorph relationships advanced by preceding anatomists on the basis of distinct data sources (mainly osteology). Interestingly, most of these relationships were never recovered in recent molecular phylogenies, such as the sister-group relationship between the Batrachoidiformes and Lophiiformes [52]–[54], Callionymoidei and Gobiesocoidei [1], [44], [66]–[68], and Mugiliformes and Atherinomorphae [24], [81]; the monophyly of the Scombroidei [98] and the non-amarsipid Stromateiformes [111]; the inclusion of the Pholidichthyiformes within the Labriformes [19], [64]; and the basal allocation of the Rhyacichthyidae and Odontobutidae as successive sister-groups to remaining Gobiiformes [76]–[78] (Fig. 5). The existence of independent anatomical evidence repeatedly favoring a same set of hypotheses discordant with the molecular topologies clearly indicates that the interrelationships of most major percomorph groups is still far from a satisfactory resolution.

The lack of basic comparative data for several anatomical systems is a major problem that hampers the production of morphology-based phylogenies of ray-finned fishes. The primary homologies (or hypotheses of homologies) of several structures are often unclear, especially among major actinopterygian groups, and the production of comprehensive, detailed studies of comparative anatomy [e.g. 10,48,114–116] is crucial to mitigate this sort of problem. The present study is an attempt to provide basic and detailed knowledge on the percomorph infrabranchial musculature, so that this anatomical system may be more satisfactorily incorporated into larger data matrices of future phylogenetic analyses. Denser sampling of percomorphs and incorporation of characters from other systems may, and likely will, reveal important phylogenetic signal in all characters herein discussed, including those which now seem to display mosaic distributions.

## Supporting Information

Table S1 Material examined.
(DOCX)
