# Correction: The Infrabranchial Musculature and Its Bearing on the Phylogeny of Percomorph Fishes (Osteichthyes: Teleostei)

**DOI:** 10.1371/journal.pone.0112600

**Published:** 2014-11-04

**Authors:** The PLOS ONE Staff

## Notice of Republication

This article was republished on October 14, 2014, to correct misnumbered sections describing the phylogenetic characters in the online version of the paper. The publisher apologizes for this error.

## References

[1] Datovo A, de Pinna MCC, Johnson GD (2014) The Infrabranchial Musculature and Its Bearing on the Phylogeny of Percomorph Fishes (Osteichthyes: Teleostei). PLoS ONE 9(10): e110129 doi:10.1371/journal.pone.0110129 25310286 10.1371/journal.pone.0110129 PMC4195711

