# Supplementary material for: The Infrabranchial Musculature and Its Bearing on the Phylogeny of Percomorph Fishes (Osteichthyes: Teleostei)
Source: PLoS One. 2014 Oct 13;9(10):e110129. doi: 10.1371/journal.pone.0110129 (PMC4195711; doi:10.1371/journal.pone.0110129)
Supplement: Table S1 — Material examined. (DOCX) [file pone.0110129.s001.docx]

**Table S1**. Material examined.

| **Order** | **Family** | **Species** | **Catalog #** | **Examined specimens** |
| --- | --- | --- | --- | --- |
| Acanthuriformes | Acanthuridae | *Acanthurus chirurgus* | MZUSP 48207 | 2 |
|  | Luvaridae | *Luvarus imperialis* | MCZ 55003 | 1 |
|  | Siganidae | *Siganus punctatus* | MZUSP 69497 | 1 |
|  | Zanclidae | *Zanclus cornutus* | USNM 348977 | 1 |
| Anabantiformes | Anabantidae | *Anabas testudineus* | USNM 393943 | 1 |
|  |  | *Ctenopoma petherici* | MZUSP 84478 | 2 |
|  | Channidae | *Parachanna obscura* | MZUSP 84470 | 2 |
| Atheriniformes | Atherinopsidae | *Atherinella brasiliensis* | LIRP 7527 | 2 |
| Batrachoidiformes | Batrachoididae | *Batrachoides surinamensis* | MZUSP 8876 | 1 |
|  |  | *Porichthys porosissimus* | MZUSP 46971 | 1 |
|  |  | *Thalassophryne nattereri* | MZUSP 47261 | 1 |
|  |  | *Triathalassothia lambaloti* | MZUSP 87217 | 1 |
| Beloniformes | Exocoetidae | *Exocoetus volitans* | MZUSP 48963 | 1 |
|  |  | *Parexocoetus hillianus* | MZUSP 103868 | 1 |
| Beryciformes | Holocentridae | *Holocentrus ascensionis ^1^* | MZUSP 60324 | 2 |
| Blenniiformes | Blenniidae | *Scartella cristata* | MZUSP 60573 | 2 |
|  | Tripterygiidae | *Enneanectes altivelis* | MZUSP 53039 | 2 |
| Caproiformes | Caproidae | *Antigonia capros* | MZUSP 71623 | 1 |
|  |  |  | MZUSP 108164 | 1 |
|  |  | *Capros aper* | USNM 327294 | 2 |
| Carangiformes | Carangidae | *Caranx latus* | MZUSP 64344 | 2 |
| Cottiformes | Zoarcidae | *Lycodes pacificus* | USNM 392993 | 1 |
| Cyprinodontiformes | Fundulidae | *Fundulus heteroclitus* | MZUSP 67017 | 1 |
|  | Rivulidae | *Hypsolebias antenori* | MZUSP 38342 | 2 |
| Dactylopteriformes | Dactylopteridae | *Dactylopterus volitans* | MZUSP 79889 | 1 |
| Elassomatiformes | Elassomatidae | *Elassoma zonatum* | USNM 112744 | 3 |
| Gadiformes | Merlucciidae | *Merluccius hubbsi ^1^* | MZUSP 80772 | 2 |
| Gasterosteiformes | Gasterosteidae | *Gasterosteus aculeatus* | MZUSP 106093 | 2 |
|  |  | *Pungitius sinensis* | USNM 336886 | 1 |
| Gobiesociformes | Callionymidae | *Synchiropus agassizii* | MZUSP 66707 | 2 |
|  | Draconettidae | *Centrodraco oregonus* | USNM 159234 | 1 |
|  | Gobiesocidae | *Gobiesox strumosus* | MZUSP 112355 | 1 |
| Gobiiformes | Eleotridae | *Dormitator maculatus* | MZUSP 65345 | 2 |
|  | Gobiidae | *Bathygobius soporator* | MZUSP 66368 | 2 |
|  | Odontobutidae | *Odontobutis obscura* | USNM 264892 | 2 |
|  | Rhyacichthyidae | *Rhyacichthys aspro* | USNM 247300 | 1 |
|  |  |  | USNM 371845 | 1 |
| Icosteiformes | Icosteidae | *Icosteus aenigmaticus ^2^* | HSU 81-305 | 1 |
| Labriformes | Cichlidae | *Cichla* cf. *piquiti* | LIRP 6317 | 2 |
|  | Labridae | *Thalassoma noronhanum* | MZUSP 48266 | 1 |
|  | Pomacentridae | *Abudefduf saxatilis* | MZUSP 36269 | 2 |
| Lophiiformes | Antennariidae | *Antennarius striatus* | MZUSP 17127 | 1 |
|  | Chaunacidae | *Chaunax suttkusi* | USNM 187848 | 1 |
|  |  |  | USNM 187852 | 1 |
|  | Lophiidae | *Lophius gastrophysus* | MZUSP 80455 | 1 |
|  |  |  | MZUSP 85811 | 1 |
|  | Ogcocephalidae | *Ogcocephalus* sp. | MZUSP 61865 | 1 |
| Mugiliformes | Mugilidae | *Mugil curema* | LIRP 7525 | 2 |
| Nototheniiformes | Nototheniidae | *Nototheniops larseni* | USNM 301728 | 1 |
| Ophidiiformes | Ophidiidae | *Raneya fluminensis* | MZUSP 61371 | 1 |
| Perciformes | Apogonidae | *Apogon americanus* | MZUSP 43144 | 2 |
|  | Badidae | *Badis corycaeus* | USNM 378881 | 1 |
|  | Bramidae | *Brama caribbea* | MZUSP 104606 | 1 |
|  | Centropomidae | *Centropomus parallelus* | MZUSP 104606 | 1 |
|  | Chaetodontidae | *Chaetodon striatus* | MZUSP 36267 | 2 |
|  | Drepaneidae | *Drepane africana* | MZUSP 84785 | 1 |
|  | Ephippididae | *Chaetodipterus faber* | MZUSP 69466 | 1 |
|  | Haemulidae | *Orthopristis ruber* | LIRP 1396 | 2 |
|  | Kyphosidae | *Girella simplicidens* | USNM 321278 | 1 |
|  |  | *Graus nigra* | USNM 289508 | 1 |
|  |  | *Kyphosus incisor* | MZUSP 44656 | 1 |
|  |  | *Microcanthus strigatus* | USNM 267047 | 1 |
|  |  | *Scorpis chilensis* | USNM 218922 | 1 |
|  | Lutjanidae | *Lutjanus analis* | LIRP 1866 | 1 |
|  | Monodactylidae | *Monodactylus sebae* | MZUSP 84858 | 1 |
|  | Nandidae | *Nandus nebulosus* | USNM 328127 | 1 |
|  | Pomacanthidae | *Centropyge aurantonotus* | MZUSP 52465 | 1 |
|  | Pomatomidae | *Pomatomus saltatrix ^2^* | MZUSP 108184 | 1 |
|  | Pristolepididae | *Pristolepis fasciata* | USNM 103105 | 1 |
|  |  |  | USNM 332697 | 1 |
|  | Scatophagidae | *Scatophagus argus* | MZUSP 102172 | 2 |
|  | Sciaenidae | *Cynoscion striatus* | MZUSP 68913 | 1 |
|  | Scombrolabracidae | *Scombrolabrax heterolepis* | USNM 187651 | 1 |
|  | Terapontidae | *Amniataba caudavittata* | USNM 173673 | 1 |
| Percopsiformes | Aphredoderidae | *Aphredoderus sayanus ^1^* | MZUSP 55046 | 1 |
| Pholidichthyiformes | Pholidichthyidae | *Pholidichthys leucotaenia* | USNM 289924 | 1 |
| Pleuronectiformes | Paralichthydae | *Paralichthys isosceles* | MZUSP 91684 | 2 |
|  | Psettodidae | *Psettodes erumei* | MZUSP 63360 | 1 |
| Scombriformes | Sphyraenidae | *Sphyraena obtusata* | MZUSP 37378 | 1 |
|  | Gempylidae | *Thyrsitops lepidopoides* | MZUSP 68473 | 1 |
|  | Istiophoridae | *Istiophorus platypterus ^2^* | USNM 229999 | 1 |
|  | Scombridae | *Euthynnus alletteratus ^2^* | MZUSP 68495 | 1 |
|  |  | *Scomber scombrus* | USNM 203841 | 1 |
|  | Trichiuridae | *Trichiurus lepturus* | MZUSP 8842 | 2 |
|  | Xiphiidae | *Xiphias gladius ^2^* | USNM 48318 | 1 |
| Scorpaeniformes | Scorpaenidae | *Scorpaena plumieri* | MZUSP 67283 | 1 |
|  | Serranidae | *Dules auriga* | MZUSP 70831 | 1 |
|  | Triglidae | *Prionotus* sp. | MZUSP 71680 | 2 |
| Stephanoberyciformes | Melamphaidae | *Poromitra capito ^1^* | USNM 250603 | 1 |
| Stromateiformes | Amarsipidae | *Amarsipus carlsbergi ^2^* | SIO 75-116 | 1 |
|  | Centrolophidae | *Psenopsis* sp. | USNM 304398 | 1 |
|  | Nomeidae | *Psenes cyanophrys* | MZUSP 67591 | 1 |
|  | Stromateidae | *Peprilus triacanthus* | MZUSP 112356 | 1 |
| Synbranchiformes | Mastacembelidae | *Mastacembelus* sp. | USNM 303353 | 1 |
|  | Synbranchidae | *Synbranchus marmoratus* | LIRP 1934 | 1 |
| Tetraodontiformes | Monacanthidae | *Stephanolepis hispidus* | MZUSP 60332 | 2 |
|  | Triacanthidae | *Triacanthus biaculeatus* | USNM 147904 | 1 |
| Trachiniformes | Percophididae | *Bembrops heterurus* | MZUSP 66958 | 1 |
|  | Uranoscopidae | *Astroscopus y-graecum* | MZUSP 46636 | 1 |
| Zeiformes | Zeidae | *Zenopsis conchifer ^1^* | MZUSP 60330 | 2 |
|  | Zenionidae | *Zenion hololepis ^1^* | MZUSP 86479 | 1 |

^1^ Outgroup taxon;

^2^ Only partially dissected.
